# Non-contact detection of ultrasound with light – Review of recent progress

**DOI:** 10.1016/j.pacs.2022.100440

**Published:** 2022-12-14

**Authors:** Jakub Spytek, Lukasz Ambrozinski, Ivan Pelivanov

**Affiliations:** a AGH University of Science and Technology, Faculty of Mechanical Engineering and Robotics, Krakow, Poland; b University of Washington, Department of Bioengineering, Seattle, WA, United States

**Keywords:** Laser ultrasonics, Optical detection of ultrasound, NDT, Ultrasound testing, Interferometry

## Abstract

In this article, we present an overview of recent progress in non-contact remote optical detection of ultrasound in application to nondestructive testing and evaluation of materials. The focus of the review is on the latest advances in optical detection that offer mature and robust field-applicable solutions for diagnostics and imaging of engineered structures. We provide a detailed description of these solutions, including their operation principles, their evolution from the previously known designs to commercial devices, and their contribution to solving the most important problems associated with non-contact optical detection of ultrasound. Several application examples are presented to demonstrate the capabilities of optical detection and provide ideas to a reader on how it can be used in practice. We also discuss the main challenges of modern non-contact detectors which have not yet been addressed, as well as the directions and prospects for their development.

## 1 Introduction

Nondestructive testing (NDT) aims to characterize material properties, often using imaging techniques, and has become an important field of science and engineering. Although various new techniques have been introduced over the past few decades, ultrasound testing (UT) remains one of the main methods in NDT. However, some disadvantages of UT methods in some cases make their application inefficient and, therefore, must be overcome. Indeed, in order to launch ultrasound (US) signals into the sample under study an acoustic coupling (i.e., a material placed between the transducer and the target) is necessary. This is not a problem when small samples are investigated, especially when the sample can be immersed into a liquid. A much more complicated situation arises when large industrial components (turbine blades or airplane wings) are inspected. Liquids such as oil or water, commonly used in these applications, not only make the inspection difficult but are also cumbersome or/and can contaminate the material under examination.

The development of coupling-free (or air-coupled) inspection methods in UT has been a target problem of multiple studies for years [1], [2]. It can be overcome using air-coupled US transducers, but the dramatic impedance mismatch between solids and air and the significant attenuation of high-frequency components in air severely limit the sensitivity and resolution available for this technique. However, it appears that both problems, i.e., the need for a coupling agent and limited bandwidth, can be overcome using laser ultrasonics (LUT). Pulsed laser radiation can be used for the excitation of ultra-broadband US signals with the bandwidth limited by the duration of the laser pulse envelope, by the spatial characteristics of the laser beam profile and optical penetration depth. Laser excitation of ultrasonic waves has several mechanisms and is generally a complex problem depending on laser radiation parameters, optical, thermal and mechanical properties of the medium, and boundary conditions [3]. A profound review of the laser excitation process is beyond the scope of this paper, but the most important cases concerning the thermoelastic regime can be found in [3], [4] and the ablative regime excitation is considered in [4], [5].

Regardless of the boundary conditions at the air/material interface, the laser excitation parameters must be restrained to qualify the technique as nondestructive. The laser pulse energy and the delivered power per unit surface area must be adjusted to ensure the thermoelastic regime of operation and avoid ablation or degradation of the surface. This requirement has motivated the research and development of new detectors with sufficient sensitivity to receive US signals of relatively low amplitude.

Note that when contact with the test material is allowed, ultra-broadband laser-generated US signals alone have a significant advantage over conventional piezoelectrically-generated signals when they are detected by well-designed broadband receivers. PVDF (polyvinylidene difluoride) transducers [6], [7], optical detectors based on diffraction gratings [8], [9], [10] and optical resonators [11], [12] have been proposed, which have shown significantly improved resolution, sensitivity and performance in NDT. A. Karabutov’s group published multiple works on the material characterization and developed an optoacoustic defectoscope [13] which greatly outperformed the conventional UT devices. Recently, contact micron-scale aperture all-optical detectors have been demonstrated for broadband US detection with sensitivity that cannot be achieved with piezoelectric transducers, especially for high frequency (greater than 20 MHz) applications [9].

Although LUT methods with contact receivers have shown a great advantage over conventional UT, there are many applications where contact between the sample and the measurement probe is not desirable or even impossible. These applications include, but are not limited to, high-temperature applications, remote sensing of radioactive chambers, large constructions (such as turbine blades), airspace and car industry NDT, metallurgy, inspection of fragile substances, etc.

Non-contact detection of ultrasound has a long history, beginning with laboratory devices and now including commercial systems for field applications. Advances have been made in both material evaluation and biomedical applications, as detailed in a series of reviews [4], [14], [15]. Seminal work was performed by J.-P. Monchalin's group [16], [17], [18], [19]. That design based on a confocal Fabry-Perot interferometer, significantly improved the probe light collection from uneven surfaces. Other approaches aimed at minimizing the effects of speckle structure include modifications of the Sagnac interferometer [20], [21], [22] and interferometer designs based on photorefractive crystals [23], [24], [25]. However, the detection sensitivity of most optical techniques does not usually approach that of a well-designed contact piezoelectric transducer.

Recent advances in the field of diode and fiber optical sources, optoelectronics and telecommunication optics have made it possible to propose new solutions and make sufficient progress in non-contact optical detection of US signals. Some solutions have only been implemented in the laboratory environment and it is too early to conclude how they may advance the remote detection. However, others have already reached a level of high technological readiness, allowing them to be used in the field.

This work does not describe the basics of laser ultrasonics and numerous laboratory designs of optical detectors, which can be found in previous reviews [10], [26]. We also do not cover picosecond laser ultrasound, as it requires separate consideration due to differences in approaches and equipment. We rather aim to review the latest advances in optical detection of ultrasound that offer mature and robust field-applicable solutions for non-contact, nondestructive evaluation of engineering structures.

## 2 Ultimate sensitivity of US wave reception

One of the key characteristics of a receiver is the sensitivity to detect mechanical vibrations, or the minimum signal (pressure, velocity, or displacement) magnitude that can be distinguished from the background noise floor. Almost every publication introducing a new detection approach first addresses its sensitivity compared to existing methods. Similarly, the main question for non-contact optical detection of ultrasound, which must be discussed among others, is whether optical detection can achieve the sensitivity of conventional piezoelectric transducers.

Most non-contact optical approaches use a portion of the light reflected from the sample surface. The larger the effective numerical aperture (NA) of the detection optics is used, the higher the reception efficiency can be achieved. The choice of NA is determined by the specific problem in which the detector is used. Since the wavelength of the probe light is on the order of 1 μm, the spot size on the detection surface varies from units to hundreds of micrometers. Additionally, the surface roughness of industrial materials reduces the light collection with decreasing NA, and sometimes the required bandwidth of the detected signals does not allow increasing the probe beam spot size on the detection surface.

In the piezoelectric approach, US transducers usually focus the probe US beam onto the sample to reach the desired lateral resolution and the depth of field. Acoustic beam characteristics can be calculated based on the transducer’s central frequency, its bandwidth and NA, and material properties.

The ultimate limit for any acoustic detector, independent of its nature, is set by the Johnson–Nyquist noise power associated with molecular thermal vibrations, as given by the well-known formula [27]:

(1) $$W_{\mathrm{Nyquist}}=4k_{B}T\Delta f=8.28*10^{-14}W,$$

where $k_{B}$ is the Boltzmann constant, T=300K and ∆f=5MHz corresponds to the bandwidth of a typical measured reference signal spectrum. The noise equivalent pressure can then be defined as:

(2) $$P_{\mathrm{Noise}}=\sqrt{\frac{{\rho \mathrm{cW}}_{\mathrm{Nyquist}}}{S}}$$

where ρ and c are the density and sound speed in the medium, and S is the surface area over which acoustic vibrations are recorded. Here, however, is a principal difference between the optical and piezoelectric approaches to define their noise floor characteristics.

In case of the optical approach, $S=S_{\mathrm{op}}$ is equal to the 1–100 μm focal spot area size of the probe optical beam. However, $S\approx S_{\mathrm{transducer}}$ in case of the focused contact transducer, i.e., it is defined by its entire reception aperture, which is usually 5–20 mm. Although such an estimate may depend on impedance matching between the sample and the transducer (including the coupling fluid), the effective reception area S of a typical transducer is still much larger than the detection laser spot. Thus, one should expect a 10–30 dB difference between the ultimate detection sensitivities of optical and piezoelectric approaches. Thus, the sensitivity of optical detection can never reach the sensitivity of contact US transducers even if their performance is ideal.

Note that it is usually very difficult to reach the noise limit in the US signal detection. Because the ultra-broadband detection is required for laser-generated US signals, US detectors are usually far from the ‘ideal’ performance. Optical detectors are broadband, and therefore their performance closer approaches the noise limit for the given reception aperture. For example, apples-to-apples comparison between a broadband PVDF transducer and the Sagnac optical detector operated in a similar frequency range was performed in Ref. [27], and a ∼10 dB difference in a noise equivalent pressure was shown between the detectors. Such difference is not critical for most NDT applications. If that noise figure could be reached for all inspection cases, it would have totally changed the NDT field and the conventional UT been entirely replaced with LUT. However, often other issues turn the detection capabilities of optical detectors down to a much worse performance compared to that described above. Below we discuss typical challenges of non-contact optical detection and attempts to overcome them in recent optical detecting designs.

## 3 Methods

### 3.1 Multi-channel random quadrature interferometer

#### 3.1.1 Design

Most non-contact optical detectors of ultrasound rely on interferometry. Its basic principle is well known [4]. Briefly, two interfering beams are necessary for the signal detection. One of the beams typically serves as a reference beam, which is usually reflected from a mirror and has an ideal phase and amplitude profile. The second beam (sample beam) is formed by the reflection of the probe light from the sample surface. An US wave introduces a displacement on the surface of the sample, which modifies parameters of the sample beam, and therefore the evolution of the intensity of the interference between the beams makes it possible to determine the temporal dependence of vibrational surface displacement caused by the US wave.

**Fig. 1:**
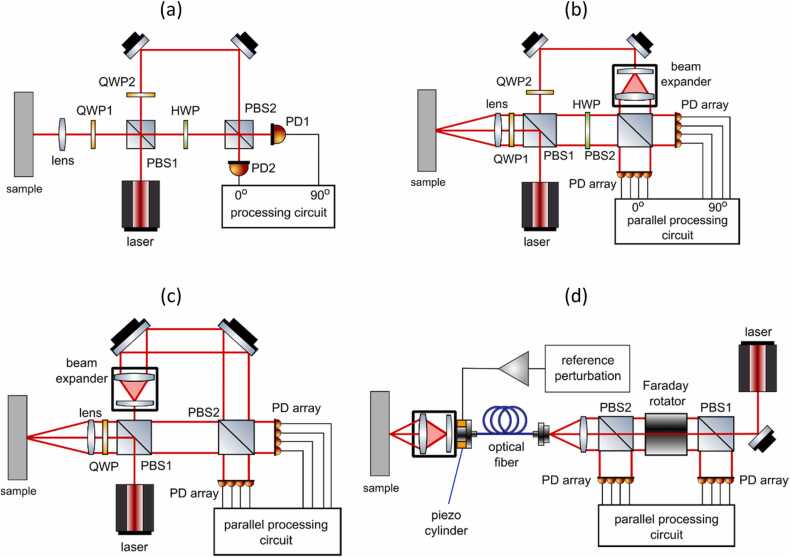
(a) Mach Zehnder interferometer [30], (b) multi-channel Mach Zehnder interferometer [30], (c) random quadrature Interferometer [30], (d) random quadrature Interferometer with multimode optical fiber delivery.

For example, the basic architecture of Mach-Zehnder Interferometer using a Quadrature Demodulation scheme is presented in Fig. 1a. The laser light emitted by the source is split into two beams using beam splitter (PBS1): a reference beam and a sample beam. The sample beam is circularly polarized using a quarter-wave plate QWP1 and then focused by the lens on the measured surface, while the reference beam is circularly polarized using a quarter-wave plate QWP2 and projected onto mirrors. The sample beam reflected from the measured surface is collected by the lens, linearly polarized, then passes through a half-wave plate (HWP) and is then mixed with the reference beam using a polarization beam splitter PBS2. The two composing linearly polarized portions of the reference beam interfere with the linearly polarized sample beam at the two outputs of PBS2 [29]. Since the two components of the reference beam have a 90-degree phase shift with respect to each other, the resulting electronic fringe signals are in quadrature. They can then be processed using a quadrature demodulation scheme to compute the surface displacement.

All figure panels are reproduced with permission from Ref. [30].

The setup shown in Fig. 1a requires both the reference and sample beams to be plane waves. A random phase distribution of speckles means that only a small portion of scattered light can contribute to the amplitude of measured signal making the interference inefficient. Rough surfaces tend to scatter the incident light, resulting in a speckled reflection beam. Therefore, most of the power of the probe beam reflected from the surface becomes unusable and the detection efficiency drops sharply. Effectively, only a single speckle should be collected to maintain phase coherence, which leads to a significant reduction of sensitivity.

To collect multiple speckles, the detection aperture size can be enlarged, and an array of photodetectors used instead of a single detector, as presented in Fig. 1b. The sample beam composed of multiple speckles is mixed with the expanded reference beam and then projected onto arrays of photodetectors [29], [30]. If the critical alignment of optics is maintained, each corresponding photodetector pair in both arrays yields a quadrature signal, which can be processed using quadrature demodulation and then summed to increase the signal magnitude. While this setup is more sensitive than the single-channel interferometer, it still requires precise alignment and is therefore not very robust.

An extension of the multi-detector principle was proposed by Pouet et al. [29], [31] in the form of Multi-Channel Random Quadrature (MCRQ) interferometer. The scheme of their system is presented in Fig. 1c, which is a simplified version of the multi-channel interferometer from Fig. 1b [29] that does not use a standard quadrature detection. The MCRQ takes the advantage of the random optical phase distribution, assuming that statistically 50 % of signals registered by photodetectors are in-quadrature and 50 % are out-of-quadrature. Therefore, if the signals from multiple photodetectors are demodulated separately and then summed together, the overall sensitivity of the measured signal remains high without the need for stabilization, since the out-of-quadrature signals will not contribute to the resulting signal. To obtain high sensitivity, two arrays of 25 photodetectors are used in the proposed interferometer. Each signal recorded by the array must be individually demodulated and processed. Relying on statistical speckle distribution to perform quadratic demodulation makes the MCRQ interferometer robust without strict optical alignment requirements.

To further improve the system’s flexibility, the MCRQ interferometer was adapted to use the multimode optical fiber delivery (Fig. 1d). In that configuration, the sample beam is projected through the fiber and then focused onto the measured surface using a lens in the optical head. A portion of the beam is then reflected from the surface and then collected by the fiber, acting as a sample beam. Part of the incident laser beam is reflected by the fiber’s end (4–5 % energy) and used as a reference beam. Moreover, a small piezoelectric actuator is attached to the end of the fiber to vary the object path between the lens and the fiber’s end. In this way, a known Doppler shift based pilot signal is added to the sample beam, which is later used during the signal processing to perform the sign correction. The reference and sample beams are combined together during the backpropagation in the optical fiber. The mixed beam travels through the first polarization beam splitter, which directs vertically polarized components into the detector array. The rest of the beam passes through an optical isolator in the form of Faraday rotator, half-wave plate and the second polarization beam splitter, and then is directed as a vertically polarized component to the second detector array. The optical isolator also protects the laser from any possible back-reflections.

**Fig. 2:**
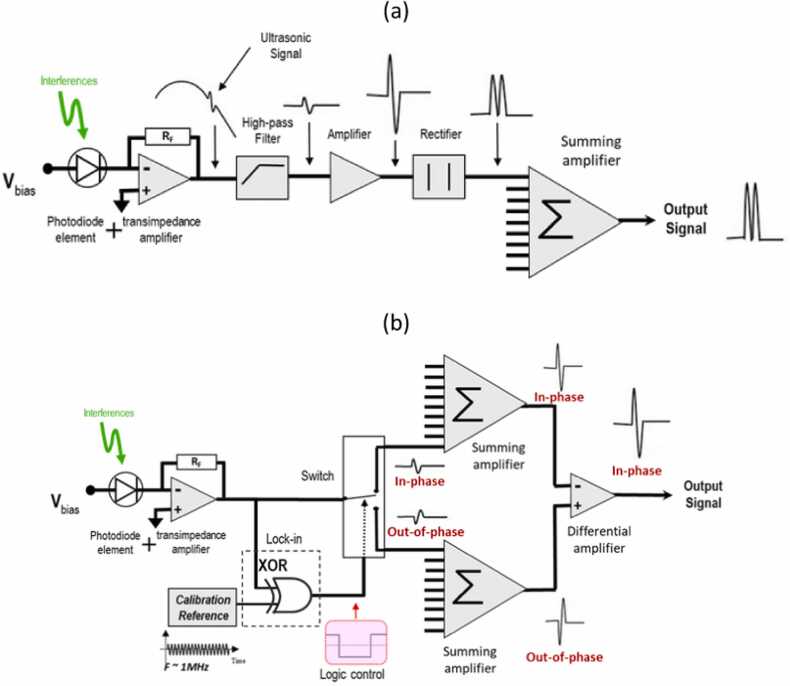
Schemes for (a) rectified demodulation and (b) linear demodulation.

Reproduced with permission from Ref. [32].

The MCRQ interferometer uses two possible demodulation schemes: a rectified demodulation, and a linear demodulation, as shown in Fig. 2. The rectified demodulation involves high-pass filtering to remove low-frequency perturbations, followed by the amplification and rectification of the signal. Assuming the random quadrature principle, all the individual signals are summed together to obtain the resulting signal. This scheme efficiently rejects background noise, but the information on the direction of displacement is lost.

The second demodulation scheme, i.e., the linear demodulation, involves a logic control, which detects the signal phase. Depending on whether the signal is in-phase or out-of-phase, the logic switches between the two summing amplifiers to add the appropriate signals constructively. In this demodulation scheme, switching is based on the low-frequency Doppler shift introduced by the piezo-ring attached to the fiber. The low-frequency signal is separated from the high-frequency signal and used to switch the summation logic circuit based on the low-frequency sign. Thus, the displacement direction of the signal can be correctly recovered.

The approach described above was commercialized by Sound & Bright (CA, USA).

#### 3.1.2 Application examples

**Fig. 3:**
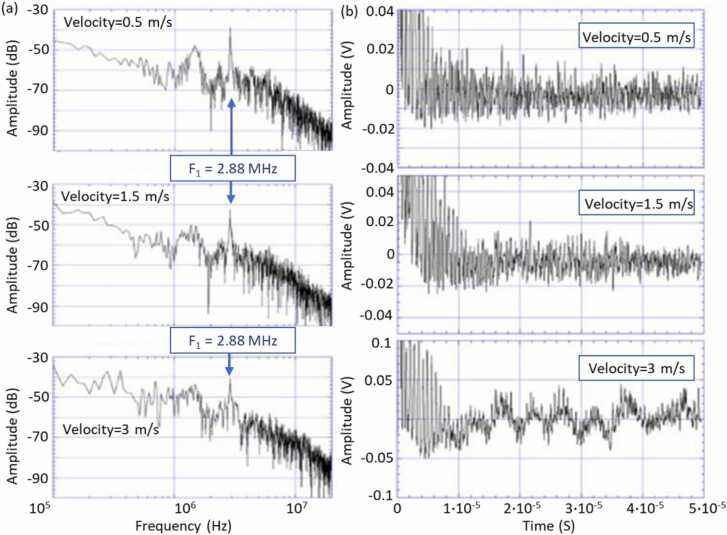
Signals measured on the surface of a rotating aluminum disc using pulsed laser excitation and the multi-channel random quadrature interferometer for the US signal reception.

Adapted with permission from Ref. [31].

One of the first applications of the multi-channel random quadrature interferometer was presented by Pouet et al. [31]. The first measurement was performed on the surface of a rotating 2 mm thick aluminum disc, which moved at a linear velocity of 3 m/s in the measured spot, in order to demonstrate the robustness of the interferometer. An Nd:YAG pulsed laser was used for the LU signal excitation and its laser spot was superimposed with the probe light. Obtained results from a single acquisition were presented in Fig. 3. In the signal spectra, a distinct resonance peak at 2.88 MHz is visible, which corresponds to twice the frequency of the zero-group velocity (ZGV) Lamb mode of the 2 mm aluminum plate. Frequency doubling of the detected ZGV resulted from the rectified demodulation scheme. A 100 kHz frequency component in the measurement with a 3 m/s velocity resulted from the disc wobbling.

**Fig. 4:**
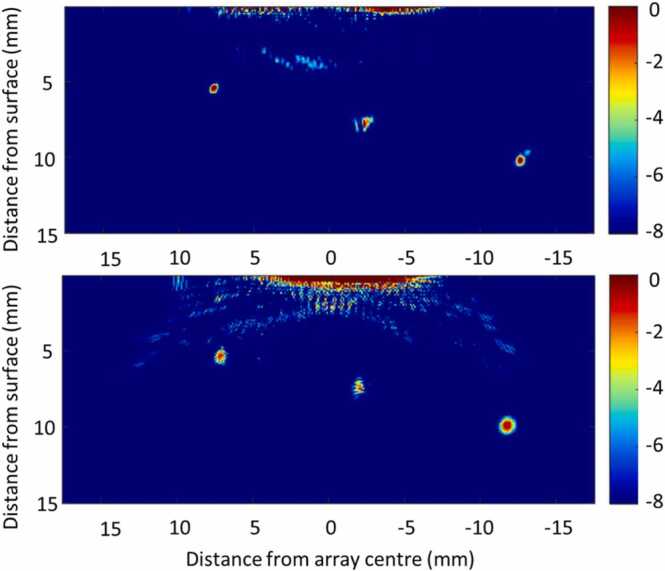
Images obtained using LIPA [33] with the multi-channel random quadrature interferometer [31] for the US signal reception. The images were obtained using shear (top image) and longitudinal (bottom image) waves.

Adapted with permission from Ref. [33].

A more recent application of the MCRQ interferometer was presented by Lukacs et al. [33] in ultrasonic imaging of a wire arc additive manufactured component Ti-6Al-4 V using a laser-induced phased array (LIPA). The sample had dimensions of 26 mm × 10 mm × 110 mm with three side-drilled holes of 1 mm in diameter located at different depths. The interferometer was placed on a translation stage to measure responses at several locations of the surface to obtain a synthetic array of detectors for the inspection. A laser source was used to provide the excitation in the same spatial locations to obtain the full matrix capture of the time response signals. A Q-switched Nd:YAG laser with a pulse width of 8 ns, 300 μJ energy and repetition rate of 1 kHz was used for the excitation of LU signals. The measured surface was polished before inspection to improve the quality of the measured signal. To further improve the signal-to-noise ratio (SNR), each captured waveform was averaged 128 times. Therefore, 14 min was required for a complete full matrix acquisition from 68 LIPA elements. Additionally, digital bandpass filters (for central frequencies of 5.5 and 6.3 MHz) were used in post-processing to further improve the SNR. Such measurement setup enabled obtaining the data, which were processed using a total focusing method (TFM) algorithm. The TFM was performed separately for longitudinal and shear waves to obtain images, revealing the locations of all three side-drilled holes, as shown in Fig. 4. Although the sample surface quality was enhanced in those measurements, the measurements were also possible with an unpolished surface with the increased number of signal averages [33].

### 3.2 Multicomponent interferometry

#### 3.2.1 Description

Most interferometers are designed to measure the out-of-plane component of vibrations, as both the incident and reflected beams are aligned close to the surface normal to maximize the signal-to-noise ratio. However, the in-plane vibration components are useful in some applications as they provide an additional information on the behavior of specific wave modes. To measure the in-plane vibration, laser beams reflected at several angles from the measurement surface should be recorded at once. Usually, optical systems using multiple laser sources aligned at different incident angles are used [34], [35], [36]. Such systems are effective, but the required alignment is not trivial and depends on the surface reflectivity. It is therefore desirable to integrate measurements of both in-plane and out-of-plane components in the same interferometer architecture [37], [38].

Optical interferometers can detect the phase of reflected light by combining the sample beam with the reference beam. The combined beams are projected on the photodetector and the interference pattern indicates the phase shift between the two beams. Wave mixing can be performed using a polarization beam splitter, as demonstrated in the previous section. For successful detection, the sample and reference beams must coincide with the wavefronts on the photodetector, and the average phase between the beams must be constant. Photorefractive crystals (PRC) [39] can record a dynamic hologram by mixing the sample and reference beams, ensuring wavefronts’ matching and automatically compensating for slow variations of phase. Moreover, multiple beams can be processed by the PRC at the same time, which is a useful feature for multiplexed interferometers [37].

**Fig. 5:**
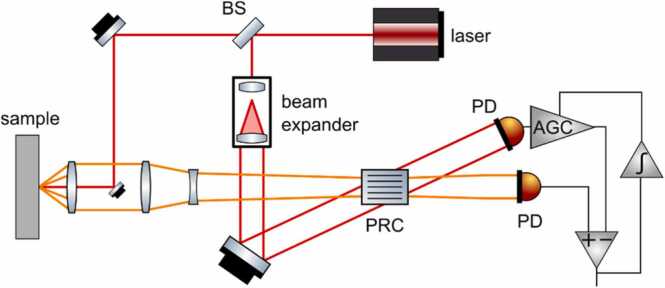
Two-wave mixing interferometer with PRC.

Adapted with permission from Ref. [40].

An example of a two-wave mixing interferometer using a PRC is presented in Fig. 5 [40]. In the given design, the laser source generates a beam, which is divided into sample and reference beams using a beam splitter. The reference beam is expanded using a beam expander and redirected towards the PRC. Simultaneously, the sample beam scattered from the object is collected using an optical lens and projected on the PRC to interfere with the reference beam. The reference and sample beams are transmitted through or diffracted by the PRC. The reference beam is diffracted in the direction of the sample beam and vice versa, yielding two interference beams. Recording the first interference beam (in the direction of the incident sample beam) using a photodetector is sufficient to compute the surface displacement. However, the second interference beam also carries small modulations from the sample beam and can be used for the noise suppression [40]. The amplitude of the second electrical signal is normalized to the amplitude of the first signal using an automatic gain control amplifier (AGC in Fig. 5), driven by the integrated output signal. The amplified signal is then subtracted from the first signal using a differential amplifier. Thus, the DC component in the output signal can be removed, and the noise intensity is significantly reduced.

**Fig. 6:**
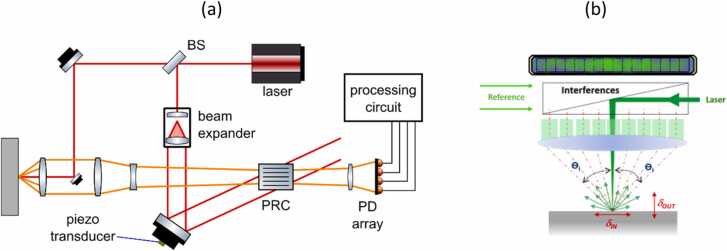
(a) Multi-component interferometer using PRC for two-wave mixing (adapted with permission from Ref. (b) [38]), (b) scheme of a linear array of photodetectors (reproduced with permission from Ref. [41]).

A two-wave mixing PRC interferometer based on the design presented in Fig. 5 was commercialized by Sound & Bright (CA, USA) and adapted to measure both in-plane and out-of-plane vibration components using a single detector [37], [38]. The architecture used in this setup (Fig. 6a) was very similar to the previous two-wave mixing interferometer, but with some differences. The information on the in-plane vibration components was contained in the light beams scattered at different angles, therefore a high numerical aperture (NA) optical lens was used to collect multiple speckles. Multiple sample beams were then simultaneously combined with the expanded reference beam at the PRC. The resulting interference beam was directed using a cylindrical lens onto a linear array of photodetectors. Each element in the array received beams corresponding to a specific reflection angle. The elements were organized symmetrically in pairs; therefore, for a n-element array, elements i and (n-i + 1) corresponded to the same angle $\theta_{i}$, according to the Fig. 6b. The signals $s_{i}$ were processed based on the assumption that the out-of-plane motion is symmetric with respect to the sample beam axis, but the in-plane motion is asymmetric. Therefore, the signal $s_{\pm i}$ can be related to both the in-plane component $u_{x}$ and the out-of-plane component $u_{z}$ using the equation:

(3) $$s_{\pm i}=\cos\left(\theta_{i}\right)u_{z}\pm \sin\left(\theta_{i}\right)u_{x}.$$

For small angles, the $u_{x}$ and $u_{z}$ components are therefore proportional to:

(4) $$\begin{array}{c} u_{z}=(s_{i}+s_{-i})/2\\ u_{x}=(s_{i}-s_{-i})/2\theta_{i} \end{array}$$

**Fig. 7:**
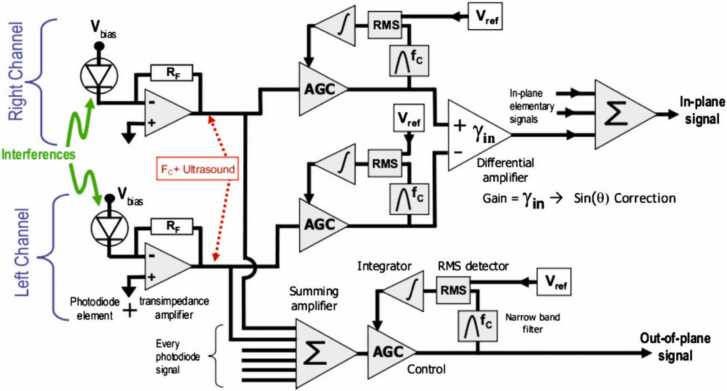
Processing circuit for multi-component interferometer.

Reproduced with permission from Ref. [38].

These equations can be implemented using a signal processing circuit, presented in Fig. 7. Signals from each detector are normalized using an automatic gain control (AGC) amplifier. For calibration, the AGC can be fed by the reference signal generated by a PZT transducer, which is mounted on the path of the reference beam. To obtain the out-of-plane displacement component, the conditioned signals are summed. For the in-plane component of the displacement, the signals are summed using a differential amplifier with a gain proportional to the incidence angle $\theta_{i}.$.

#### 3.2.2 Application examples

**Fig. 8:**
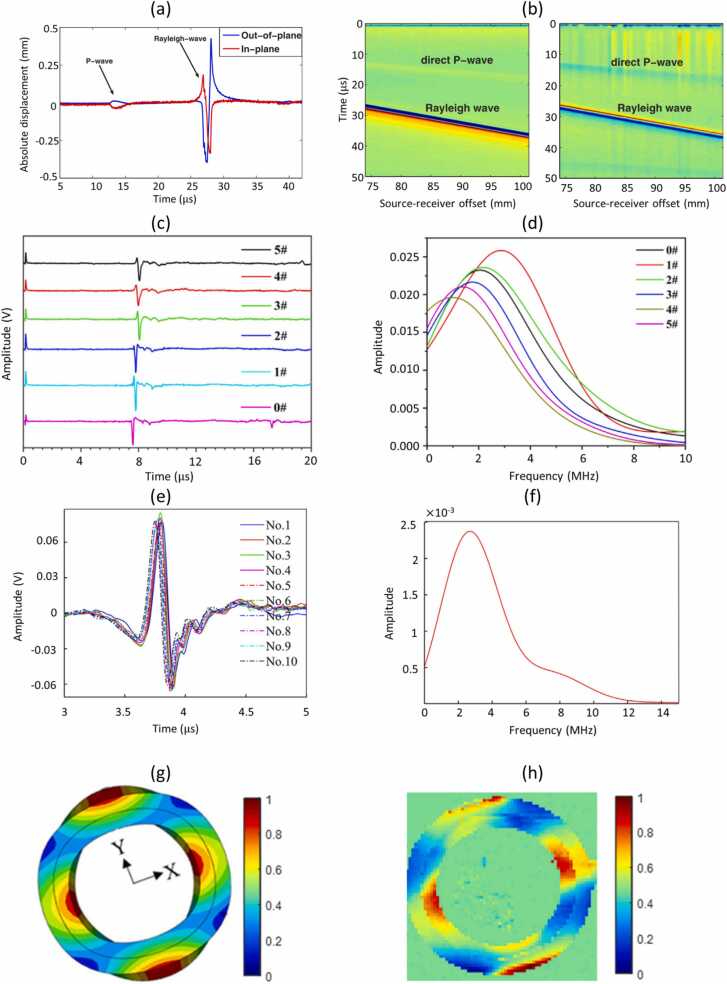
(a) Time responses of in-plane and out-of-plane Rayleigh wave and (b) line scan of Rayleigh wave – out of plane (left) and in-plane (right) components (reproduced with permission from Ref. [38]); Rayleigh wave measurement results for different titanium alloy samples: (c) time waveforms and (d) frequency spectra (reproduced with permission from Ref. [42]); (e) Rayleigh wave measurement results for different locations together with (f) exemplary Rayleigh wave spectrum (reproduced with permission from Ref. [43]); qualitative comparison of modal displacement maps at 40 kHz frequency for the composite piezoelectric cylinder: (g) numerical simulation and (h) experimental (reproduced with permission from Ref. [46]).

Application of the two-wave mixing PRC interferometer described above was presented by Blum et al. [38] for the measurement of in-plane and out-of-plane components of Rayleigh waves in an aluminum block. The waves were excited using a 250 mJ, 20 Hz pulse repetition rate Nd:YAG laser source focused to a 4 mm diameter spot at a 77 mm distance from the receiver. Measured signals were averaged 500 times and bandpass filtered between 300 and 900 kHz. The relative amplitudes of in-plane and out-of-plane components matched the theoretical calculations, while the phase shift showed a little error due to the difference in circuit frequency response for both vibration components. Examples of measured signals are presented in Fig. 8a. The setup was also used for the line scanning to obtain surface wave fields, which are presented in Fig. 8b.

Chen et al. [42] used the two-wave mixing PRC interferometer for the estimation of alpha-phase volume fraction in the titanium alloy based on the velocity, amplitude and peak frequency of Rayleigh waves. US waves were generated using light pulses from an Nd:YAG laser with a 12 ns pulse duration and a 5 mJ energy focused to a line with the length around 10 mm. The distance between the excitation and detection points was set to 22 mm, and received signals were averaged 128 times. Examples of acquired signals and their corresponding spectra are presented in Fig. 8c and d respectively.

Chen et al. [43] used the same detector to record laser-generated Rayleigh waves for the detection of subsurface defects using the evolution of the wave phase. The authors indicated that a possibility of measuring both the in-plane and the out-of-plane wave components separately was vital to the presented method, as otherwise the phase of the signal could not be correctly estimated. A Nd:YAG pulsed laser with a 1064 nm wavelength was used for the wave excitation. A 12 ns, 5 mJ energy laser pulse was focused to a 15 mm-long line source. Examples of measured signals averaged 200 times are presented in Fig. 8e. The bandwidth of measured Rayleigh waves was mainly concentrated in the range from 2 MHz to 4.5 MHz, as shown in Fig. 8f.

Stampfli et al. [44] and Newacheck et al. [45] used the two-wave mixing PRC interferometer to concurrently measure in-plane and out-of-plane displacement components in a multiferroic composite structure subjected to sinusoidal electric AC field (30 kHz or 32.5 kHz). To increase the SNR, the surface was sanded to remove marks and scratches and then polished to near mirror finish prior to measurement using the interferometer [45]. The amplitudes of measured in-plane and out-of-plane displacements were smaller than 150 nm and 60 nm respectively. The interferometer was scanned using a 2D translation stage to obtain a high-resolution (0.1 mm) spatio-temporal map. In the related publication [46], in-plane and out-of-plane displacement fields were measured and used for modal analysis of multiferroic composites. The obtained experimental results matched well the results of numerical simulations, as demonstrated in Fig. 8g and h.

Garcia de la Yedra et al. [47] used the two-wave mixing PRC detector for the measurement of acoustic emission from cracks occurring during laser cladding of Stellite alloy layers onto the steel substrate.

### 3.3 Speckle knife edge detector (KED)

#### 3.3.1 Design

**Fig. 9:**
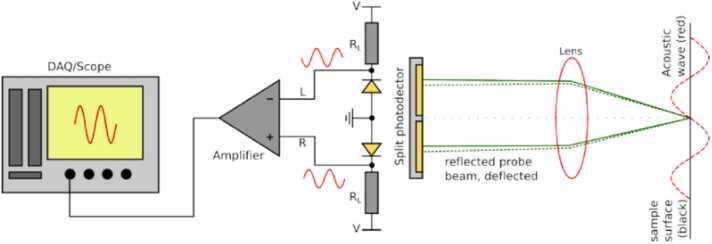
Knife-edge detection scheme using a position-sensitive split photodetector.

Reproduced with permission from Ref. [48].

Knife-edge detection (KED) is a non-interferometric technique for the measurement of vibrations based on the optical beam deflection. Surface vibrations result in the alteration of the incident optical beam direction when the beam size is smaller than the ripple spacing [16]. The KED enables measuring the deflection of the reflected beam using the knife edge positioned in front of the receiving photodetector. Therefore, the change in the amplitude registered by the photodetector is proportional to the deflection of the beam, as it can be partially obscured by the knife edge [16]. The KED can also be implemented using position-sensitive photodetectors, e.g. a split photodiode [48], as shown in Fig. 9. The beam tilt induced by US vibration causes a shift of the speckle projected on the photodetectors, resulting in the amplitude change registered by each photodetector. The output from both detectors is fed to a differential amplifier, which produces a signal proportional to the amplitude of the surface vibration. However, the KED works only if the surface is optically smooth [49]. For optically rough surfaces, the reflection is usually diffuse, resulting in a speckle pattern, i.e. a combination of bright and dark spots. For the KED, the speckle pattern results in a significant drop of the measured signal amplitude.

**Fig. 10:**
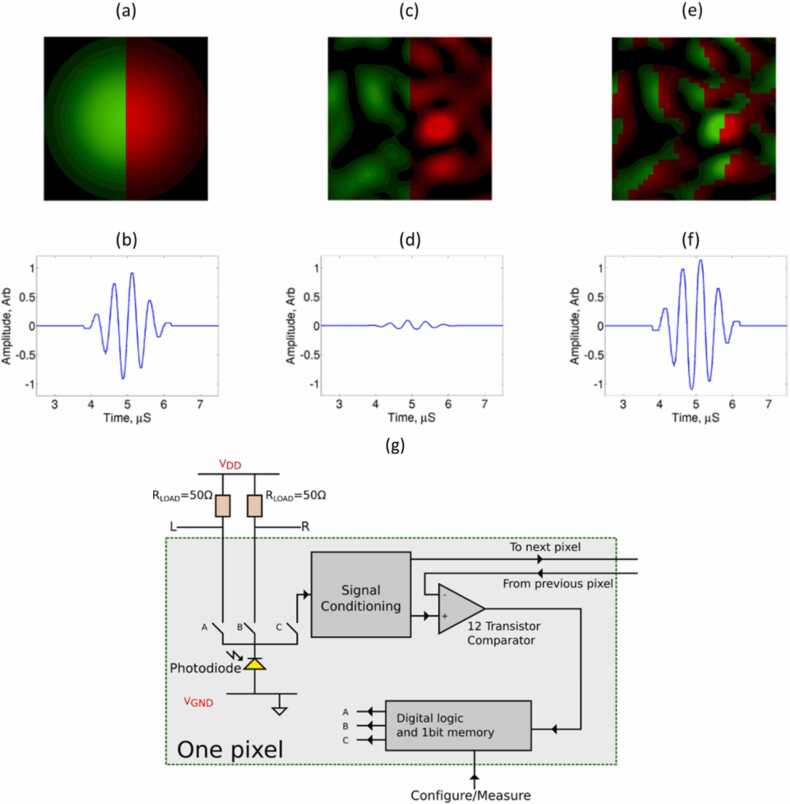
Comparison between SKED and KED: (a) light field collected from a smooth surface and received with KED and (b) detected US signal; (c) light field collected from a rough surface and received with KED and (d) detected US signal; (e) light collected from a rough surface and received with the configurable KED and (f) detected US signal; (g) processing circuit for each pixel in SKED CMOS array.

Reproduced with permission from Ref. [48].

The limitation of KED for probing optically rough surfaces has been addressed by a speckle knife edge detector (SKED) [48], [50], [51]. The SKED takes advantage of synchronous motion of different bright-dark edges in the speckle pattern by using a CMOS array of photodiodes to detect and evaluate the individual speckles in the image. The operating principle of SKED is presented in Fig. 10. Each photodiode generates the signal proportional to the light intensity, according to the amplitude distribution of the speckle pattern.

Fig. 10a shows a pattern for the specular reflection (for an ideal surface), close to Gaussian shape, which is shifted proportionally to the magnitude of surface vibration. To properly resolve this motion, signals from the half of diodes (green area) are summed and fed to the negative input part of differential amplifier (similar to the scheme in Fig. 9), and signals from the other half (red area) are fed to the amplifier’s positive input part. This setup works functionally as a standard KED shown in Fig. 9 with more diodes used. Direct application of a fixed differential processing for the specular reflection results in a well-resolved signal, as demonstrated in Fig. 10b.

If the inspected surface is rough, the sample beam is reflected diffusively, which results in multiple speckles on the CMOS array, as shown in the Fig. 10c. Applying the same processing scheme as in the case of specular reflection results in a signal presented in Fig. 10d, which has much lower amplitude compared to that in Fig. 10b. The amplitude drop occurs because dark and bright speckles cancel each other.

The solution to this problem offered by SKED is to tune the CMOS array to the speckle pattern by identifying individual speckles and using some of them as positive and some of them as negative signals, which is done using the following algorithm. Assuming that the speckle pattern shifts horizontally, for each pair of two neighboring pixels, the circuit checks which of the two receives a higher light intensity. If the right-hand side pixel has the higher intensity, the output from the right-hand detector is processed as positive (‘red’) signal. If the left-hand side pixel has the higher intensity, then the right-hand side pixel is processed as ‘green’ (negative) signal. As a result, the CMOS array is adjusted to the speckle pattern resulting from the measured beam, as shown in Fig. 10e. The sums of positive and negative signals are then subtracted to obtain the signal proportional to the surface vibration. Therefore, the amplitude loss from the speckle pattern is compensated, as it is shown in Fig. 10f.

In the latest reported version, SKED consisted of a CMOS array with 32 × 32 elements [51]. The size of each pixel in the array was 59 μm × 59 μm, and 43 % of the array area was photosensitive. A signal from each pixel was processed using a circuit presented in the Fig. 10g. The SKED processing circuit can operate in two configurations. In the ‘configure’ mode, it can automatically adjust the matrix signs to the interference pattern using the algorithm described above. The sign of each pixel (either positive or negative) is stored in memory. In the ‘measurement’ mode, the comparator is turned off and the current from the photodiodes is directed through pre-programmed paths.

According to Ref. [48], setting up the SKED to a particular speckle pattern takes about 0.1 μs, and the measurement can be completed within 0.5 μs. The SKED bandwidth is limited by the capacitance of photodiodes and the parasitic impedances from switches and traces. For a configuration where half of photodiodes are negative and half are positive, the − 3 dB bandwidth was approximately 16 MHz. However, signal reception up to 82 MHz was reported in Ref. [48].

A more recent version of SKED, named SKED2 [48], improved the overall performance by introducing some changes into the design. Switches inside the circuit were simplified to reduce parasitic impedance effects. Moreover, a second comparator was added to check if a single-pixel amplitude in the ‘configure’ mode exceeds the value of a reference current. If the photodiode receives too little light, it is not connected to the output in the ‘measurement’ phase. In this approach, diodes that do not contribute to the signal are excluded. This approach can improve the amplitude of the measured signal. Turning off the non-contributing diodes by increasing the reference current resulted in an increase in the signal amplitude by about 20 %.

Note that the presence of parasitic impedances and different amplitudes of the signals at different pixels reduce the coherence of individual pixels during their summation. Thus, some decrease in SNR compared to a single ideal detector with an equivalent aperture should be expected at a minimum surface roughness. However, the described SKED principle demonstrates a valuable improvement in detection of US vibrations from rough surfaces.

#### 3.3.2 Application examples

The effectiveness of SKED was demonstrated in detection of laser-generated Rayleigh waves, in particular for spatially resolved acoustic spectroscopy (SRAS) [51]. The SRAS used the excitation of surface waves at a predefined wavelength using a grating mask for the laser illumination. By recording LU signals at a fixed distance from the source and computing the LU signal frequency, the local propagation speed can be determined. The map of in-plain distribution of material elastic properties could be obtained by scanning the sample at different propagation directions.

**Fig. 11:**
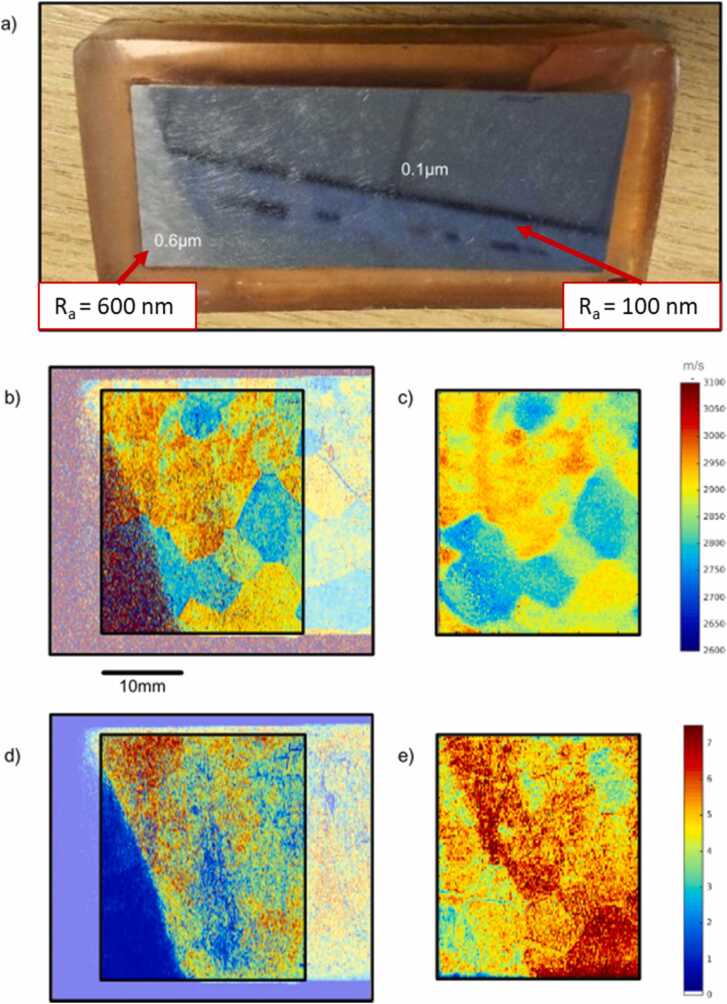
Results of spatially resolved acoustic spectroscopy (SRAS) for evaluating the granular structure of a Ti6246 block (a) with different levels of roughness. SRAS maps obtained using (b) KED and (c) SKED detectors; maximum amplitudes of locally measured signals using (d) KED and (e) SKED.

Reproduced with permission from Ref. [51].

Patel et al. [51] performed a SRAS measurement on a Ti6246 block with surface roughness ranging from $R_{a}=100$nm to $R_{a}=600$nm, as shown in Fig. 11a. LU signals were excited with a Q-switched 1064 nm Nd:YAG laser (600 μJ pulse energy, 8 ns pulse duration and 1 kHz repetition rate). The SKED detection was performed with a 1.5 W, 532 nm CW laser. The SRAS maps obtained in the experiments are demonstrated in Fig. 11b–d. Fig. 11b, d show maps of local velocity and maximum amplitude respectively for standard KED system, while Fig. 11c, e show the same maps for the advanced SKED system. The overall quality of measurements obtained with the SKED has improved, especially in the area with $R_{a}=600$nm (‘triangular’ area in the bottom-left corner). The maximum signal amplitude measured by the KED is near zero in the region with $R_{a}=600$nm, while signals obtained with the SKED provide usable information.

The detection of Rayleigh waves in steel samples using SKED was demonstrated by Lee et al. [52]. Surface acoustic wave signals were generated by a Q-switched Nd:YAG laser emitting 10 ns pulses with a pulse energy of 10 mJ. A line excitation scheme was used by focusing the laser beam on the sample surface with a cylindrical lens after beam expansion. Surface waves were measured at different distances from the excitation source, in the range from 9 to 25 mm. The measured signals had an 8 MHz bandwidth which were used to determine the thickness of the surface-hardened layers based on the computed dispersion curves.

### 3.4 ‘Signal diversity’ Laser Doppler Vibrometer (sdLDV)

#### 3.4.1 Design

Heterodyne interferometers offer a slightly different approach compared to homodyne interferometers described in previous sections. Heterodyne interferometers are based on the measurement of surface vibrations using the principle of light beam modulation due to the Doppler effect [53], therefore the instruments are often called Laser Doppler Vibrometers (LDV). The displacement signal can be reconstructed from optical phase modulation, while the surface velocity is obtained from the optical frequency shift. However, the optical frequency of the probe laser beam is too high for direct demodulation, so the heterodyne interferometry principle has to be used.

**Fig. 12:**
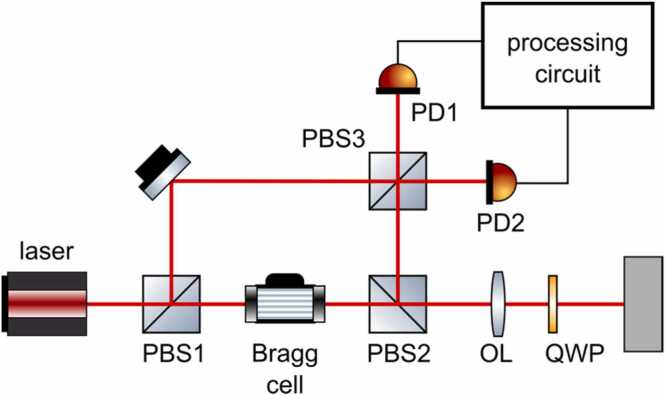
Scheme of a heterodyne laser doppler interferometer.

Reproduced with permission from Ref. [57].

An exemplary scheme of the heterodyne interferometer in Mach-Zehnder architecture is presented in Fig. 12. The laser source is split using a polarization beam splitter PBS1 into sample and reference beams. The sample beam is directed through a Bragg cell, which shifts the optical frequency by the frequency of the input control signal. The sample beam is first directed towards PBS2 and then projected through an optical lens and a quarter-wave plate on the measurement surface. The reflected beam is then collected by the lenses, directed towards PBS3, mixed with the reference beam to produce interference, and then projected onto the detector (often a balanced photodiode). Heterodyne light signals registered by the photodetector, $i_{\det}\left(t\right)$, contain modulations resulting from surface vibrations, according to the following equation [53]:

(5) $$i_{\det}\left(t\right)=I_{\mathrm{DC}}+\mathrm{icos}(2\pi f_{0}t+\phi_{m}(t)),$$

where $I_{\mathrm{DC}}$ is the DC component, i is the AC amplitude, $f_{0}$ is the frequency of the Bragg cell and $\phi_{m}(t)$ is the phase modulation resulting from the surface displacement. The signal $i_{\det}\left(t\right)$ needs to be processed using an analog demodulator to extract the surface vibration, which can be performed using different demodulation schemes [53].

Heterodyne interferometry is a mature technology, and there are many commercially available instruments provided by several manufacturers, including Polytec, Dantec, Ometron and Optomet. Unfortunately, LDVs are very sensitive to dark speckles, especially when measurements are performed on a rough surface. Due to surface vibration, the speckle pattern may evolve, resulting in variations of the measured signal. The fluctuation of speckles influence the Doppler signal through the amplitude or phase modulation [54]. Amplitude modulation can lead to random amplitude drops, resulting in so-called ‘drop-outs’ [55], [56]. Phase modulation may result in spurious frequency components called pseudo-vibrations. These effects significantly limit the capabilities of LDVs.

The issue of signal drop-outs was addressed in new Polytec® vibrometers by applying a principle of signal diversity [55], [56], [57]. The occurrence of drop-outs is random due to fluctuations in the speckle pattern and can be described in terms of statistical probability. If a vibration is measured from two or more statistically independent signals, the probability of drop-out occurring on each signal at the same time is the product of probabilities for each channel. Thus, the overall drop-out probability is significantly reduced. Each measured signal should be processed by a separate photodetector. The required statistical independence of signals can be achieved in several ways [57]:

• Combination of different polarizations (orthogonally polarized), especially in case of diffusively reflecting surfaces, where the reflected light is randomly polarized.
• Application of spatial separation, e.g., different parts of the probe beam or beams measured by different apertures can be processed independently.
• Application of modal separation, meaning that different orthogonal beam modes can be processed separately, as the energy of the speckles is distributed into multiple modes.

**Fig. 13:**
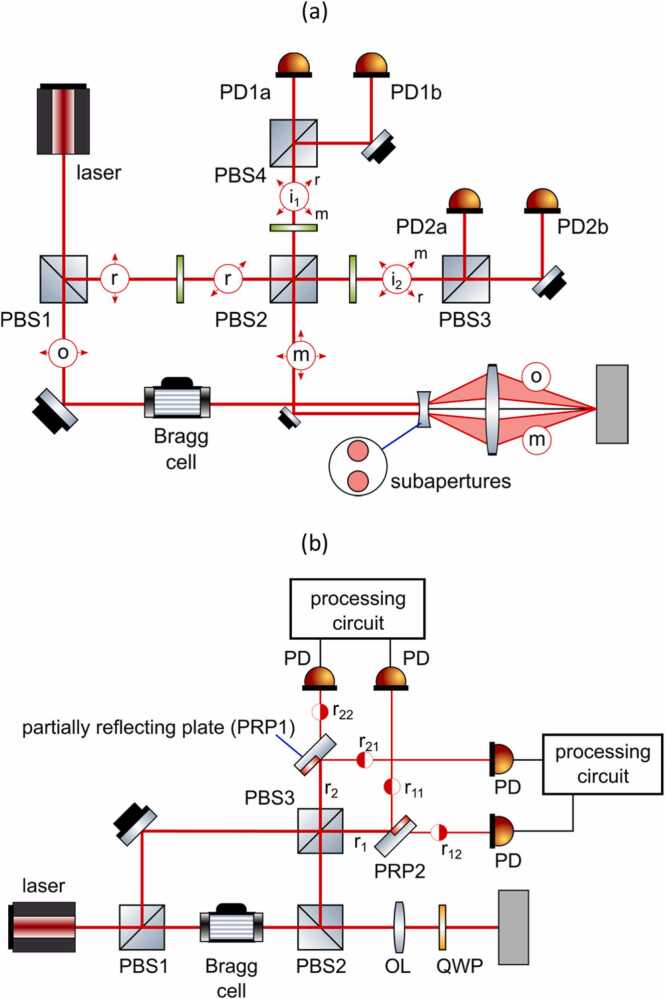
Scheme of laser Doppler vibrometer with (a) polarization diversity and (b) spatial diversity.

Reproduced with permission from Ref. [57].

A prototype of a heterodyne interferometer using polarization diversity was demonstrated by Dräbenstedt et al. [55]. The interferometer was based on the Polytec RSV-150 architecture, and its scheme is presented in Fig. 13a. The sample beam *o*, separated using PBS1, is directed through the Bragg cell and then projected onto the measurement surface using an optical lens. The reflected sample beam m is received by a second sub-aperture and transmitted parallel to the emitted beam. Therefore, it is not necessary to use a beam splitter to separate incident and reflected beams, and both orthogonally polarized components of the reflected sample beam can be used. The beam m is then simply reflected by a mirror and mixed with the reference beam r at PBS2. Each polarization component of the beam m is overlaid with the orthogonally polarized component of the reference light r, and then rotated by 45 degrees. Effectively, two separate beams are created ($i_{1}$ and $i_{2}$), each containing both the reference r and the reflected sample beam m components, which are able to interfere. These two signals are then received by separate pairs of balanced photodetectors (PD1 and PD2) and each signal is demodulated separately, creating two independent signals. It is noteworthy that the use of sub-apertures for transmitted and reflected beams leads to a decrease in the energy of collected light (by about 25 % of the nominal amplitude). It is partly compensated by the use of both polarization components.

In order to combine signals from different independent channels, each of them is independently demodulated and then multiplied by a weighting factor based on the power level of the frequency-shifted carrier signal, RSSI, which works as a signal quality indicator. Therefore, if a signal is poorly measured due to the dark speckle, its RSSI value is low, and less weight is assigned to it. The combination of signals from different measurement channels is performed according to the following weighting scheme [56]:

(6) $$s_{\mathrm{com}}\left(t\right)=\frac{\sum_{i=1}^{N}a_{i}\left(\mathrm{RSS}I_{i}\right)s_{i}(t)}{\sum_{i=1}^{N}a_{i}\left(\mathrm{RSS}I_{i}\right)}$$

where $s_{i}\left(t\right)$ is a signal measured at i-th channel, $s_{\mathrm{com}}\left(t\right)$ is the combined signal, and $a_{i}\left(\mathrm{RSS}I_{i}\right)$ is a weighting function taking $\mathrm{RSS}I_{i}$ from i-th channel as an argument. The $a_{i}\left(\mathrm{RSS}I_{i}\right)$ is strictly increasing to provide a higher weight to a signal with higher quality.

The spatial diversity can be achieved by dividing the aperture into several sub-apertures [55], [57], similarly as in the polarization diversity case, or by using separate apertures [56]. The exemplary scheme utilizing the spatial diversity, demonstrated by [57], is presented in Fig. 13b. The scheme is very similar to the heterodyne interferometer presented in Fig. 12. After the interference of reference and sample beams at PBS3, the resulting beams $r_{1}$ and $r_{2}$ are spatially separated using two partially reflecting plates PRP1 and PRP2. These plates pass one part of the beam, while reflecting the other part, resulting in four new beams: $r_{11}$*,* $r_{12}$*,* $r_{21}$*,* $r_{22}$. The two pairs of beams are then received by PD detectors and these two resulting signals are processed using the same combination method as in the previously described polarization diversity scheme.

#### 3.4.2 Applications

**Fig. 14:**
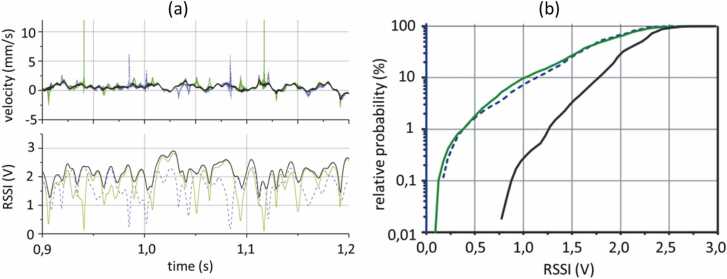
Illustration of the polarization diversity principle for signals recorded from the surface of a rotating aluminum disc using LDV. (a) Green and blue curves correspond to velocity (top panel) and RSSI values (bottom panel) for separate channels from two polarization components of the sample beam. The combination of the two signals using diversity principle is plotted with the black line. (b) Probabilities of a drop-out in signals from separate channels (green and blue curves) as a function of RSSI voltage, compared to the drop-out probability of the combined signal (black curve).

Reproduced with permission from Ref. [55].

A prototype of heterodyne interferometer using polarization diversity was used to measure vibrations of an aluminum disk rotating at 20 RPM [55]. The polarization diversity setup allowed for using two independent channels, and they were analyzed separately as well as their weighted sum. The measured signal bandwidth was up to 6.6 kHz, and the signals were sampled at 40 kHz rate. The exemplary waveforms are presented in Fig. 14a. Green and blue curves show velocity and RSSI values for separate channels from two polarization components. As evident from the figures, if the RSSI is close to 0, significant signal spikes appear in the velocity plot, corresponding to signal drop-outs. However, the combination of the two signals given by Eq. (6) (the black line) allows to eliminate drop-outs, as there is no instance when both signals have low RSSI. Fig. 14b demonstrates the signal drop-out probability as a function of the RSSI voltage. While the probabilities for individual channels are similar (green and blue lines), the probability for the combined signal (black line) is significantly lower. The signal diversity principle was commercialized by Polytec, although the exact types of diversity used within their system are not disclosed.

### 3.5 Sagnac Interferometer

#### 3.5.1 Design

Apart from the ‘classical’ two-beam interferometers discussed above of which the designs are constantly being improved, there is an additional Sagnac-type (or ring-type) interferometer, which has been in the shadow of others for decades. It has been recently reconsidered due to the development of fiber-optic technology that was essential for its proper functioning. This type of optical interferometers was not included in previous reviews and, therefore, we provide a more detailed description of its principle here.

**Fig. 15:**
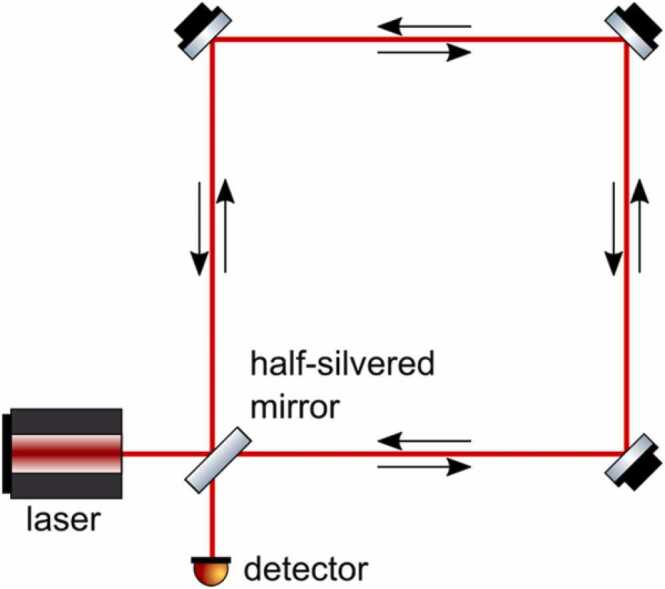
Sagnac Interferometer for the detection of system rotation.

The Sagnac interferometer, named after French physicist Georges Sagnac, represents a circular path along which two interfering beams propagate in opposite directions (Fig. 15). If the setup has no rotation, the position of interference fringes is stationary, whereas their position is shifted according to the angular velocity of the system. This effect has found important application in laser gyroscopes and metrology [58]. The Sagnac effect, for example, helps in synchronizing global navigation systems that are affected by the Earth’s rotation. There is an idea of using the Sagnac interferometer in detecting gravitational waves [59] because this could potentially serve a much simpler solution with a tremendously smaller detector footprint compared to currently exploiting Michelson Interferometer with gigantic 4 km arms.

However, as it was noticed later, if a temporally short vibration is applied to the mirror located in the right bottom corner in Fig. 15, the detection signal will be non-zero although the interfering beams still propagate the same path without applied vibrations. Indeed, the vibration causes the same change in the path of both interfering beams, but the change is introduced at different time moments defined by the lengths of optical paths to the mirror (or the lengths of the interferometer arms). When the beams bounce off the mirror and propagate back to the detector, the difference in total propagation paths is compensated due to the same total propagation path for both beams. Thus, the intensity $I\left(t\right)$ of the modulated signal on the detector is proportional to the difference in the vertical displacement $D_{z}\left(t\right)$ of the mirror measured over a small difference ∆t in time, i.e., the vertical component of the vibration speed $v_{z}\left(t\right)$:

(7) $$I\left(t\right)∼\frac{∆D_{z}(t)}{∆t}\approx \frac{dD_{z}\left(t\right)}{\mathrm{dt}}=v_{z}\left(t\right),$$

where $∆t={(l}_{\mathrm{long}}-l_{\mathrm{short}})/c$ (c – is the speed of light, $l_{\mathrm{long}}$ and $l_{\mathrm{short}}$ are the lengths of the interferometer arms).

**Fig. 16:**
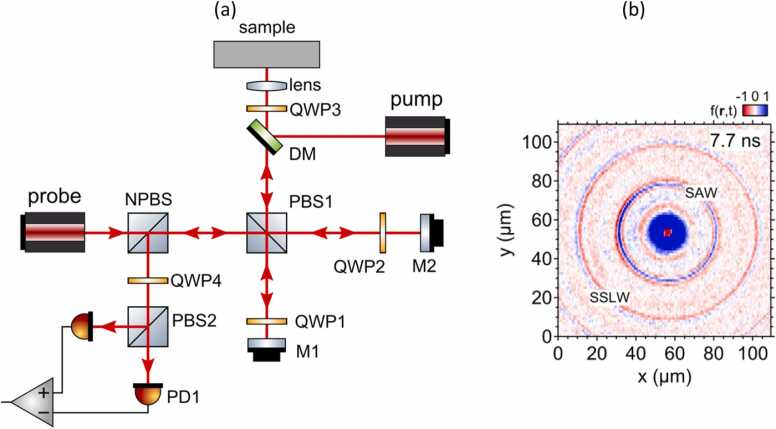
(a) Gigahertz-range Sagnac interferometer scheme (reproduced with permission from Ref. [60]), (b) the result of SAW full-field measurement obtained using the Sagnac interferometer. Reproduced with permission from Ref. [61].

In 2006, Tachizaki et al. [60] demonstrated the design of a conventional Sagnac interferometer capable of measuring surface acoustic wave (SAW) signals up to 1 GHz. The scheme of the interferometer is presented in Fig. 16a. In that design, the sample beam generated by the probe laser and linearly polarized at 45 degrees was split into horizontal and vertical beams using a polarization beam splitter PBS1. Both beams were reflected by mirrors M1 and M2 and projected onto the sample surface. The arm between PBS1 and mirror M2 was slightly longer than the arm between PBS1 and M2, therefore the second beam arrived at the surface after the first one. Both beams were then reflected from the surface, collected by the lens, and then followed a different path through the PBS1. For example, if a first beam traveled from the probe to the sample through PBS1-M1 arm, on the way back it traveled through the PBS1-M2 arm. In the end, both beams reflected from the surfaces were directed towards the quarter-wave plate QWP4 and the PBS2, where they interfered on a balanced photodetector. QWP4 introduced a shift of 90 degrees between the two beams to set the interferometer in a state of maximum phase sensitivity. Due to the time shift between the beams, the measurement of surface displacement on the sample was based on the difference between the signals reflected from the surface at two time instants. A femtosecond laser with a wavelength of 830 nm operating at a repetition rate of 76 MHz was used for the probe beam. In the presented system, the LUS was excited using a pump laser operating at the second harmonic of the probe beam (415 nm). During the measurement, the pump laser pulses were synchronized with the probe laser pulses. Therefore, the change between the two reflected probe beams was defined by the phase shift of the signal due to the out-of-plane displacement of the surface perturbed by the pump laser. The presented Sagnac interferometer was designed for a detection of high frequencies. The upper frequency limit was inversely proportional to the duration of the probe optical pulse. The authors indicated that the theoretical frequency range of up to 50 THz was possible.

The presented Sagnac interferometer was used in several applications, including measuring GHz-range surface waves in metamaterials [61] (an exemplary snapshot of the wavefield is presented in Fig. 16b) or recording zero-group velocity Lamb waves up to 10 GHz frequency in a silicon-nitride plate [62].

As can be seen, the first applications of the Sagnac interferometer were limited to the reception of US waves of very high frequency. Indeed, the detection bandwidth BW of the Sagnac interferometer is:

(8) $$\mathrm{BW}\approx \frac{1}{∆t}=\frac{c}{l_{\mathrm{long}}-l_{\mathrm{short}}}.$$

For example, a 1 m difference in path lengths results in a BW=300MHz. Reducing the bandwidth to the lower megahertz range (typical to conventional NDT studies) would result in a few tens of meters of Sagnac loop, which would be difficult to implement using open space optical components.

**Fig. 17:**
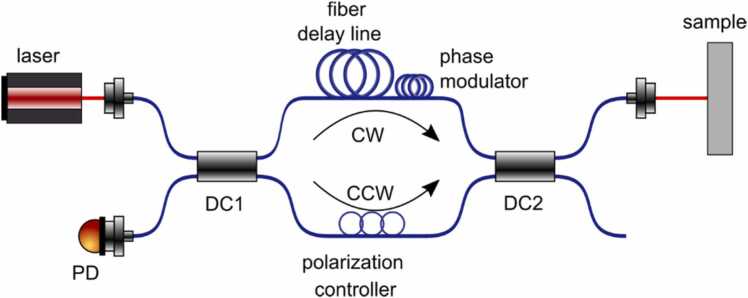
Schematic of Sagnac interferometer based on the original design by Bowers et al. [63] in 1982.

Reproduced with permission from Ref. [63].

Bowers et al. [63] in 1982 (see Fig. 17) first introduced the Sagnac Interferometer using a fiber-optic design for the detection of ultrasound vibrations, where the tens of meters Sagnac loop length was no longer an issue. The study proposed an asymmetry in the Sagnac interferometer halves so that beams propagating into different directions of the Sagnac loop arrived at the measurement surface with the delay from each other (determined by the delay line) which was compensated on the way back from the surface to the detector when both beams complete the entire Sagnac loop. Unfortunately, the existing fiber-optic technology that existed at the time did not allow the Bowers’s innovation to work properly. The clockwise and counterclockwise beams were not encoded properly, and the single mode fibers used in the system were sensitive to environmental vibrations.

**Fig. 18:**
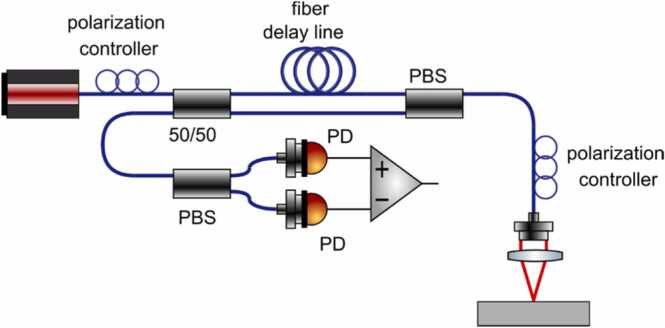
One of the Sagnac interferometer designs proposed by Alcoz et al. [65] in 1997.

Reproduced with permission from Ref. [65].

In 1997, Alcoz [64], [65] (see Fig. 18) introduced several advanced modifications of the Sagnac interferometer for the detection of US signals with the design using only polarization-maintaining (PM) fibers that allowed to properly encode clockwise and counter clockwise propagating beams with independent circular polarizations of light in the Sagnac loop. A polarization controller working as a quarter-wave plate was proposed to automatically rotate the paths of the propagation beams. The only drawback of the proposed design was in the use of the in-fiber quarter-wave plate. Although the Alcoz’s patent declared that the in-fiber polarization controller would work as a quarter-wave plate rotating the beams, to date, there are still no polarization controllers capable of changing the light polarization inside the PM fiber. In addition, a piece of fiber between the polarization controller and the detection head was used to propagate signals polarized along both slow and fast fiber axes, although the speed of light depends on the polarization in the PM fiber. Thus, an unbalanced difference for right and left circularly polarized signals was introduced, which dramatically reduced the sensitivity of the proposed method, especially in the case of using low-coherent probe light sources. However, the main reason that the method developed by Alcoz was forgotten for years is what it was only patented without clear demonstration of its advantages in literature.

**Fig. 19:**
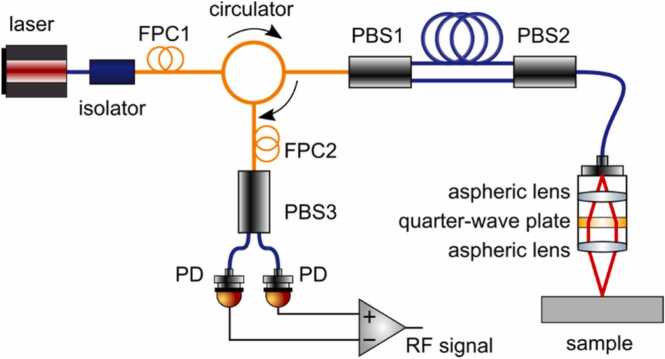
Sagnac interferometer design proposed by Pelivanov et al. [27] in 2014.

Reproduced with permission from Ref. [27] .

In 2014, Pelivanov, Buma and O’Donnell revisited the Sagnac technology to develop a fiber-optic interferometer independently of Alcoz. The proposed design, as shown in Fig. 19, was free of the drawbacks of previous approaches. In particular, modern fiber-optic components combining PM and single-mode (SM) fibers were used to build the interferometer. Instead of using circularly polarized light, the design [27] used linearly-polarized radiation which was controlled at all steps by two in-fiber polarization controllers. A quarter-wave plate to automatically switch the light propagation direction within the Sagnac loop after its reflection from the target was placed inside the detection head within the collimated beam so that the equivalence of propagation paths of the interfering beams was not violated. The detection head consisted of aspheric lenses minimizing aberrations and maximizing light reception back into the PM fiber. However, the major factor influencing the light reception from the sample surface is the surface roughness, reducing the amount of the recorded light from nearly 100 % down to the fraction covered by the transducer effective NA (in the worst situation of surface isotropic scattering).

The linear polarization design also allowed the balanced detection to remove the non-birefringent component from the detected signal and reduce the common noise. A little footprint super-luminescent diode (SLD) was used as a stable low-coherent source, which provided more than 50 dB dynamic range of the detection and did it insensitive to in-fiber reflections due to the short coherence length. The only constraint on the coherence length in the proposed design is that it should be larger compared to variations in the topography of sample surface to provide coherent reflections within the area covered by the probe beam. Finally, a fully PM-fiber design with an in-fiber polarization controllers (OZ Optics) made of short piece of SM fiber was commercialized by LuxSonics Inc.

The modern fiber-optic design makes the Sagnac interferometer competitive with other designs and even outperforms them in several characteristics. Indeed, most designs discussed above require a reference arm, which adds complexity into the system and requires complex stabilization techniques. In the Sagnac approach, both interfering beams come from the sample surface and no reference arm is required. Because the same surface is used to reflect both interfering beams that are reflected from the same target spatial point, the thermal lensing effect introduced by the pump beam is almost eliminated for frequencies above a few kHz. In addition, in interferometers that use a reference arm, it is necessary to “protect” it from environmental vibrations. The Sagnac interferometer is free of noise coming from the reference arm. However, in two-beam interferometers, the most serious negative effect is driven by the principle difference between the ideal mirror-like reflector of the reference arm and the speckle-based reflection introduced by most surfaces of untreated materials. This leads to the dramatic decrease of sensitivity of conventional designs; and most innovations in two-beam interferometer designs (discussed above) were aimed to compensate for the speckle effect. In contrast, the phase retardation effect has no influence on the Sagnac interferometer made of PM fibers. Indeed, optical fields of both Sagnac beams are the same without surface vibration. PM fibers propagate both fields with minor distortions and make them interfere in the second polarization controller. Only the difference between the fields of the two beams is important, and not the phase compound of the individual fields. Note that the amount of detected light is still affected by surface roughness. This problem can potentially be solved by increasing the probe light source power up to 300 mW (damage limit for PM components), but unfortunately, stable low coherent 1550 nm sources with the power of more than 40 mW are currently not available on the market.

The Sagnac loop automatically equalizes the paths of the interfering beams, which will be stable under any environmental conditions except for the rotation of the interferometer. This maximizes the interference to the theoretical limit because interfering beams travel exactly the same distance and meet at exactly the same time. This allows using a very compact, low coherent SLD source to greatly reduce the effects of parasitic interferences within the interferometer itself and minimizes speckles when examining regular rough surfaces.

A unique feature of the Sagnac design is the ability to quickly tune the detection bandwidth over an extremely broad range (from kHz to GHz) by changing the fiber length within the same device and without additional adjustment. This allows to apply the interferometer for both low and high-frequency measurements. Using PM fibers makes it possible to focus the probe beam spot to the diffraction limit, which enable its potential application for spectroscopy of microscale objects, such as single cells.

Traditionally, the noise figure of interferometers is compared to the shot noise of photodetectors, which is also an intrinsic characteristic of the detector. Such estimations are important and represent the ultimate sensitivity of the detector, but these estimations do not indicate how large the detection signal is compared to the ambient noise. Indeed, Johnson-Nyquist noise defines the thermal noise power, which is the minimum acoustic signal power that can be detected regardless the type of the detector used. The authors of Ref. [27] , estimated the Sagnac noise power and found that it was about 12 dB greater than the Johnson-Nyquist noise power. That figure overestimated the noise factor of the interferometer itself since it included all the electronics in the signal path. Note that an unvarnished aircraft composite surface with less than 1 % reflectance was used as the target for evaluation. Later, the noise figure was further improved to around 8 dB.

Despite the fact that the noise figure of the Sagnac detector is quite small, the overall NEP of the detection system is still modest compared to contact US probes because of the small detection area (probe light focusing spot). For a mirror-like sample surface, the probe beam diameter can be increased, and the overall optical power can be increased to significantly reduce the NEP. However, the main problem with LU inspection of industrial materials is that their surface is usually quite rough, which requires a focused probe beam and high NA optics to efficiently collect backscattered light. On the other hand, the larger the NA, the shorter the depth of field. The trade-off between a high NA and sufficient depth of field is a subject for future optimizations of confocal systems, such as the Sagnac detector.

A disadvantage of the current Sagnac principle is the difficulty of measuring the absolute displacement without calibration, which is sometimes impossible due to highly variable light reflection properties of the target. Another disadvantage is its sensitivity to a possible depolarization of the probe light induced by its reflection from the target surface.

#### 3.5.2 Applications

**Fig. 20:**
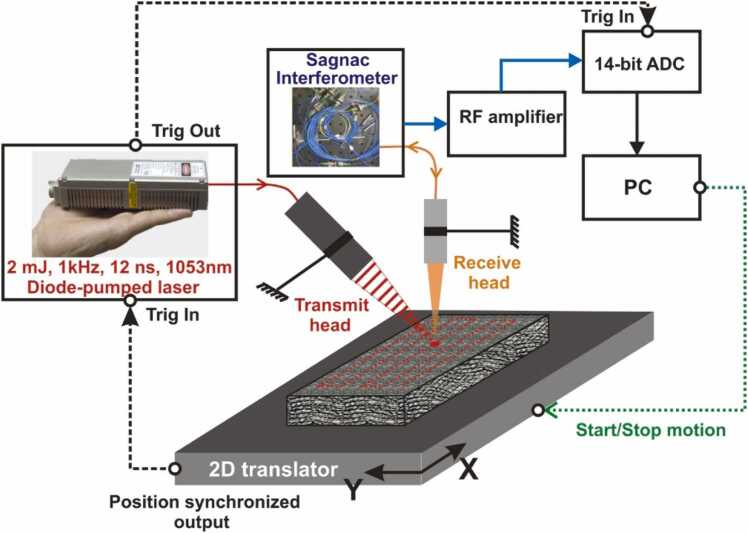
LU scanner with a Sagnac interferometer on receive [67].

Reproduced with permission from Ref. [67].

**Fig. 21:**
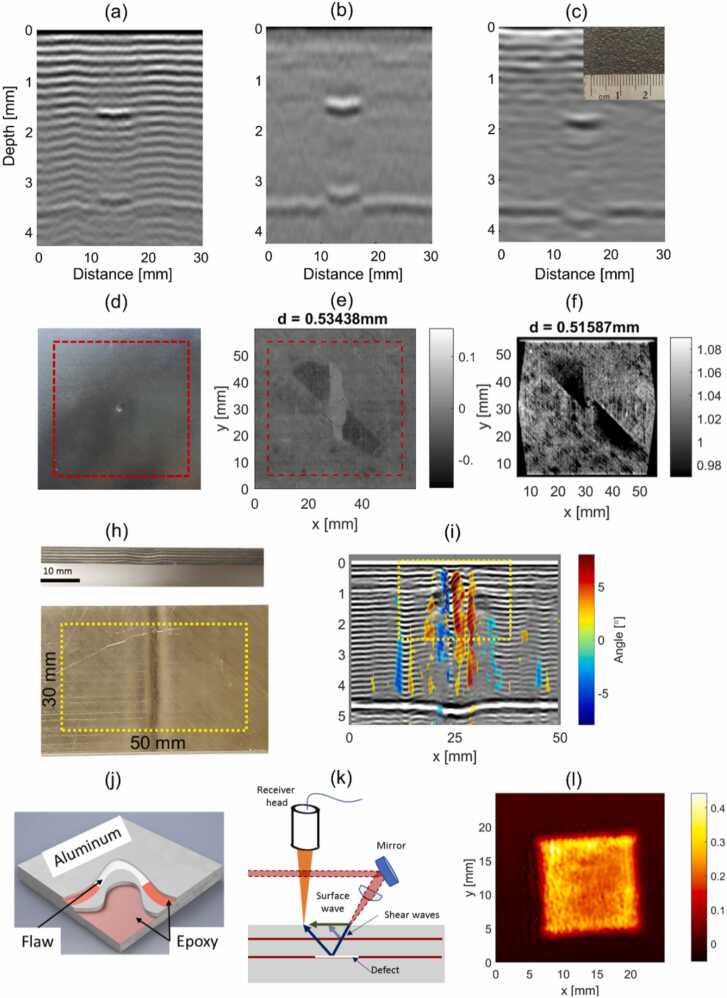
Summary of recent results obtained using the LU scanner with a Sagnac interferometer on receive [27]. LU scan of a 19-ply fiber reinforced graphite-epoxy composite sample from the front side, regular quality surface: (a) - full-bandwidth (1–10 MHz), (b) – low-pass (1–5 MHz) filtered to remove the regular composite structure from the image. (c) LU scan of the same sample from its opposite (very rough) surface. Demonstration of the impact damage detection [68] (d) – photograph of the impacted sample, (e) – LU C-scan at the depth of 0.5 mm from the sample surface, (f) C-scan obtained from X-ray tomograph. Visualization of wrinkles and local ply angle orientation [69]: (h) - photograph of wrinkled sample, (i) – LU image of the sample structure overlapped with the locally computed ply angle. Evaluation of disbonds in adhesively bounded aluminum plates using laser-generated shear acoustic waves [70]: (j) – diagram of the sample, (k) – measurement diagram, (l) – LU C-scan at the depth of plate bounding, indicating a Teflon inclusion in the structure.

Reproduced with permission from Refs. [27], [68], [69], [70].

A laser-ultrasound scanner with a Sagnac interferometer on receive (see Fig. 20) was first introduced in by Pelivanov et al. in [27]. An improved sensitivity of the Sagnac detector allowed using low energy fiber or diode-pumped ns lasers with about a 1 mJ pulse energy (compared to other systems using tens and even hundreds of mJ). Such lasers have compact footprints with possibility of operation at kHz pulse rates, which make the LU system working as fast as conventional contact ultrasound systems, at that enabling a fully non-contact approach with an unprecedented resolution. The low pulse energy and a smooth Gaussian beam shape produced by the diode-pumped solid state (DPSS) laser [67] allows to avoid overheating of the target surface even for highly absorbing graphite/epoxy composites enabling the thermoelastic regime of LU signal generation. Another advantage of using the DPSS laser type is in its ability to operate at variable repetition rates with a small (less than 2 ns) jitter in triggering. Thus, a position-based trigger can be utilized at scanning where the trigger signal comes from the translator. This allows LU scanning without stopping, which dramatically reduces the scanning time. As such, LUT NDT of aircraft composites was demonstrated in Ref. [67] in a single-shot regime with a 100 μm resolution at 100 mm/s scanning rate (see Fig. 21a,b).

The reduced sensitivity of the Sagnac detector to surface roughness was demonstrated in Ref.[66] , where the detection was performed from an extremely rough (∼ 200 μm mean height) surface. Despite the reduced quality of images and very blurred structural signal, the defects were still well detected (Fig. 21c).

The broadband nature of generated LU signals and their non-contact detection with a high spatial resolution made it possible to not only detect the flaws, but to visualize the material structure, which in turn enabled new imaging capabilities not available to reach with conventional UT. Consequently, the LUT scanner was then demonstrated in the evaluation of material porosity [71], imaging of heat [72] and impact damage [68] (see also Fig. 21d-f), and wrinkles [69] (see also Fig. 21 h,i) in aircraft composites. Authors [70] modified the scanner to receive shear waves (see Fig. 21k) and used it to inspect adhesion in aluminum sandwich plates (see Fig. 21j-l). A possibility of LU signal generation and detection at the same spot was used in Ref [73] for the excitation of zero group velocity waves which were shown to be very sensitive to variations in the system structure.

### 3.6 Air-coupled all-optical akinetic sensor

Direct optical vibration sensors rely heavily on the surface quality to perform successful measurements. Despite the significant improvements to the interferometric techniques, described above, there are surfaces that are very hard to measure using optical sensors – materials with high roughness, such as concrete, living tissue, or materials with highly light-absorbing surfaces. Conventional non-contact vibration measurements use air-coupled (AC) transducers that are applicable to various surfaces by measuring waves leaking into the air from inspected materials.

Conventional AC transducers may use piezoelectric, capacitive or PVDF sensors. The main limitation for manufacturing AC receivers is a large impedance mismatch between air and most of the solids that can be used as piezoelectric materials or membranes. In addition, the AC transducers are usually highly directional, have a very narrow bandwidth and use relatively large aperture to achieve a reasonable sensitivity.

To address the limitations of conventional AC transducers, an optical membrane-free sensor was proposed by Xarion Laser Acoustics GmbH [74], [75], [76]. The sensor offers functionality similar to an AC transducer, but with the added benefits of wide bandwidth, small size and omnidirectionality. The principles and applications of the akinetic sensor is described in this section.

#### 3.6.1 Description

**Fig. 22:**
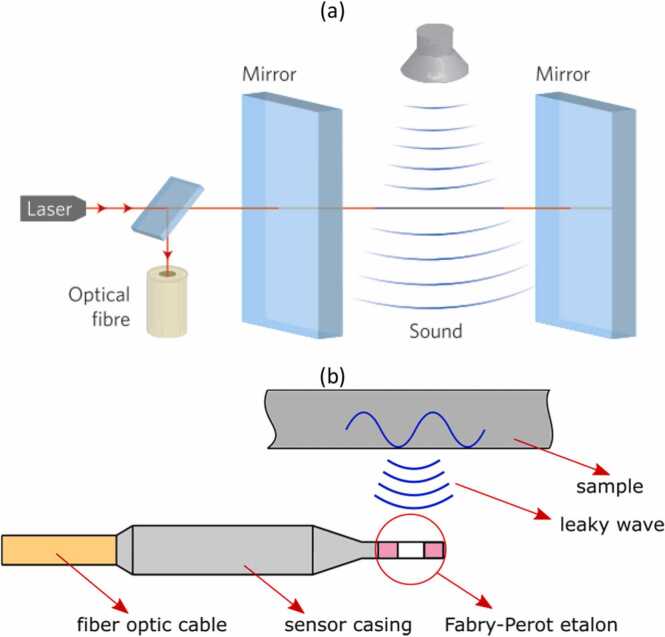
(a) Scheme of Fabry-Perot etalon used by Xarion in an optical akinetic sensor. Reproduced with permission from Ref. [75]. (b) Diagram showing a typical application of the sensor in the detection of leaky waves.

The all-optical akinetic sensor is based on a Fabry-Perot (FP) interferometer architecture, as demonstrated in Fig. 22a [74], [75], [76]. An ideal FP cavity was designed to transmit most of light intensity at a specific wavelength. At a fixed wavelength, the transfer function of the FP interferometer depends on the distance between mirrors, as well as on the reflectivity of the mirrors and the refractive index of the medium. If an US wave passes through the cavity, the refractive index of the medium is modulated by the wave. Therefore, the reflected light intensity measured by a photodiode is also modulated in proportion to the instantaneous density change caused by the propagating wave. The FP sensor operation principle can be described based on the transfer function of the ideal FP resonator by the Airy function [74]:

(9) $$\mathrm{TF}\left(q\right)=1-\frac{1}{1+F\sin{\left(\frac{q}{2}\right)}^{2}}$$

where F is the finesse coefficient, depending on the mirror reflectivity, and q is the round-trip phase shift. The phase shift q depends on the laser wavelength λ, the distance d between mirrors, and the refractive index n:

(10) $$q\left(n\right)=\frac{4\pi \mathrm{nd}}{\lambda }$$

In the given sensor, the distance between the mirrors and wavelength are fixed. Therefore, the transfer function depends on the refractive index, which can be influenced by the acoustic waves propagating between the mirrors.

The optical akinetic sensor [74], [75] consisted of the FP cavity with a 2 mm distance between mirrors. The cavity was enclosed, waterproof, and capable of measuring leaky US waves in a proximity to the surface (as shown in Fig. 22b). The bandwidth of the sensor was determined by the diameter of probe laser beam. If the acoustic wavelength was less than the beam diameter, then the measured signal was affected by averaging over the areas of high and low pressure. Therefore, the bandwidth of optical akinetic sensor is in the megahertz range. On the other hand, the practical limitation of the akinetic sensor bandwidth results from the high attenuation coefficient of ultrasound waves in air, about 160 dB/m at 1 MHz [75]. In practice, only waves below 1 MHz can be efficiently recorded.

The advantage of the optical akinetic sensor compared to traditional air-coupled transducers is the elimination of one air-solid high-impedance interface when measuring leaky waves. The sensor exhibits maximum sensitivity in a plane perpendicular to the axis of the laser source with a nearly uniform directional response profile. Amplitude drops for certain directions may be caused by the sensor casing that blocks the acoustic path [74]. The sensor has a frequency-dependent angular directivity pattern in the plane of the laser source, i.e., for 1 MHz waves the − 6 dB drop occurs at approximately 9 degrees, and for 5 MHz - at 5 degrees.

#### 3.6.2 Application examples

**Fig. 23:**
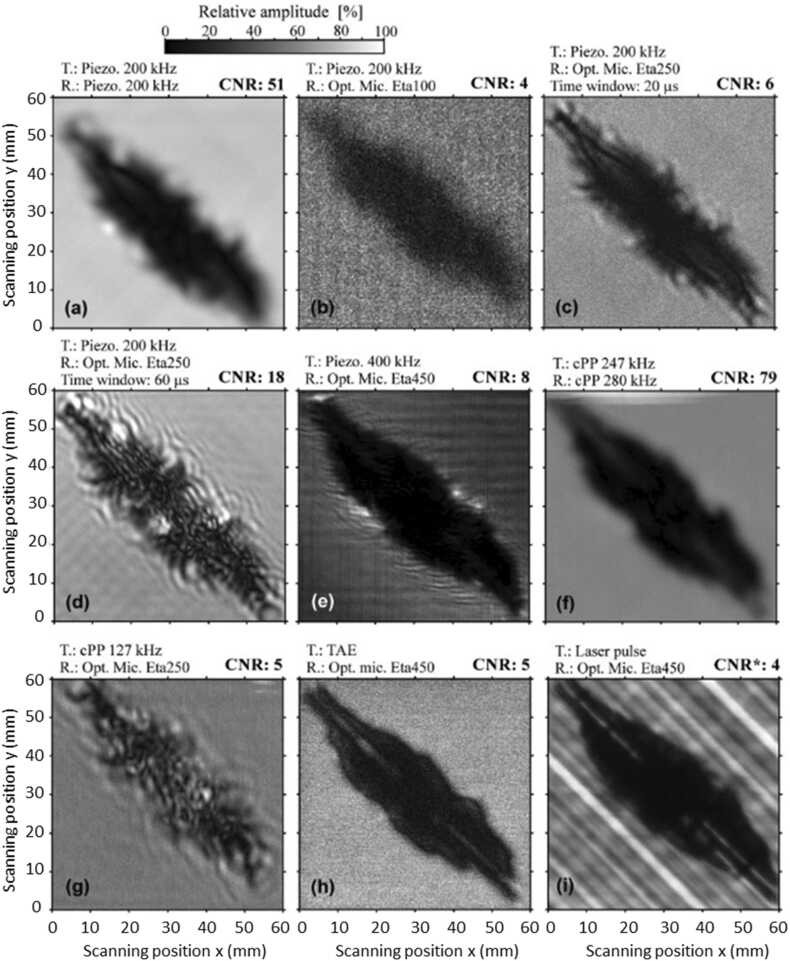
Exemplary results of a through-transmission imaging of a CFRP sample with a delamination using different configurations of transmitter (T) and receiver (R): (a) air-coupled (AC) piezo transducer operated at a T/R 200 kHz frequency, (b) 200 kHz AC transducer as T and optical microphone Eta100 as R, (c,d) 200 kHz AC transducer as T and optical microphone Eta250 as R with time windows 20 and 60 μs on received signals respectively, (e) 400 kHz AC transducer as T and optical microphone Eta450 as R, (f) piezocomposite transducers (cPP) with a 247 kHz frequency as T and with a 280 kHz frequency as R, (g) cPP with a 127 kHz frequency as T and optical microphone Eta250 as R, (h) thermoacoustic emitter (TAE) as T and optical microphone Eta450 as R, (i) laser pulse excitation (a second harmonic of Nd:YAG, 8 mm beam diameter) and optical microphone Eta450 as R.

Reproduced with permission from Ref. [77].

The optical akinetic sensor can be used in the same way as traditional air-coupled transducers. Rus et al. [77] used Xarion optical microphones Eta100, Eta250 and Eta450 in comparison with different NDT systems, including air-coupled piezoelectric transducers, cellular polypropylene transducers, laser-generated ultrasound, thermoacoustic emitter, immersion testing, phased array UT testing, X-ray radiography and thermography. The inspected sample was a 150×100×2.1 mm laminate with delaminations caused by impact. Different through-transmission tests configurations (with the US emitter located on one side of the sample and the receiver located on the other side) were performed to obtain images of the delamination, as shown in Fig. 23. The US images were evaluated in terms of resolution and contrast-to-noise ratio (CNR). Compared to other NDT systems used in the study, optical microphones provided higher spatial resolution due to the relatively small size of the detector. However, the CNR of matched piezo-transducers was significantly higher. The best results in terms of image resolution were obtained when the optical microphone was used together with high-frequency or broadband waves excited laser-ultrasonically. None of the air-coupled ultrasonic (ACU) methods could characterize the interior of the damaged area, compared to radiography or immersion methods. Overall, optical microphones were evaluated as effective tools for quick inspection and offered higher spatial resolution compared classical US transducers when the microphones were combined with laser ultrasound signal excitation.

**Fig. 24:**
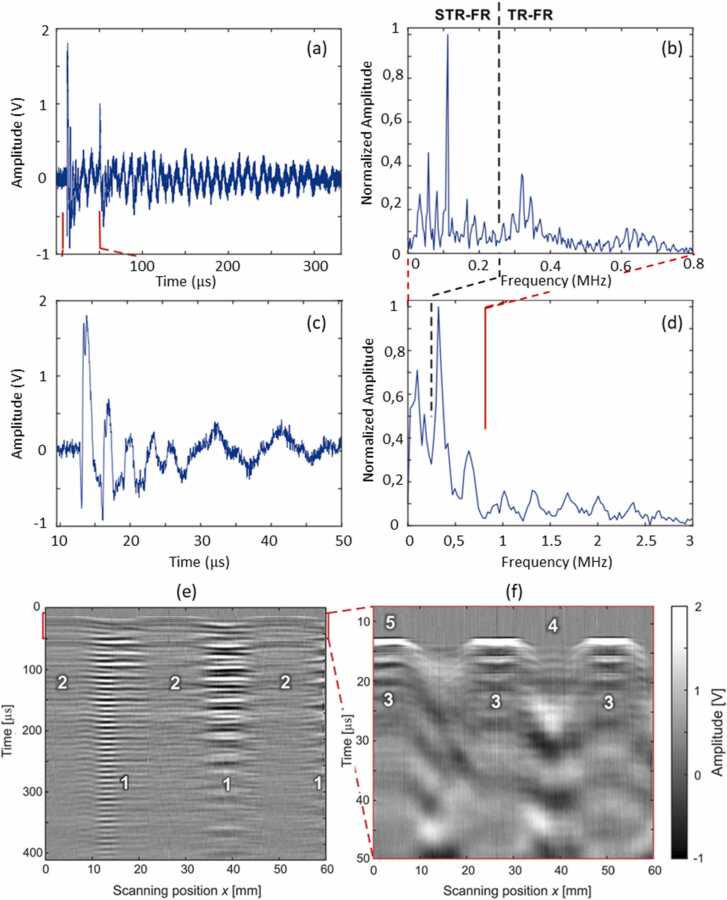
(a) Exemplary time waveform excited using LU and measured using optical microphone at the undamaged location of the CFRP and (b) the frequency spectrum of the signal. (c) Short time interval of the signal indicated by red lines and (d) the spectrum of the short time interval. (e) Exemplary B-scan obtained from the CFRP sample with visible defects indicated by number 1 and (f) short time interval of the B-scan (indicated by red lines).

Reproduced with permission from Ref. [79].

The combination of laser-ultrasound with the optical microphone on receive was presented by Rus et al. [78], [79] for applications involving local US resonance spectroscopy of the carbon fiber reinforced polymer composites. Measurements were performed for estimating local material properties from the frequency spectrum, enabling detection of defects [79] or thickness measurement [78] . Both pump and probe were scanned over a certain area, obtaining useful signals up to 3 MHz without averaging. As a result of processing in the frequency domain, maps of local thickness and damage were computed (see Fig. 24).

Haindl et al. [80] used the optical microphone as a photoacoustic wave sensor in a system, which combined optical coherence tomography (OCT) and photoacoustic microscopy (PAM) – OCT-PAM [80]. The akinetic sensor was used for measuring leaky photoacoustic waves in the PAM system. The PAM excitation could be transmitted through, and both devices were positioned on the same side of the sample to measure waves in the reflection mode.

## 4 Summary and prospects

This paper reviewed recent developments in the field of optical detection of ultrasound vibrations, with the focus on addressing some of the key issues associated with the application of LUT techniques in the field. Key areas of improvement included new schemes for measuring both in-plane and out-of-plane components of vibration, reducing the sensitivity of optical detection to surface roughness, and increasing its stability to environmental noise. Various ideas have been proposed based on the use of an array of optical detectors and improved signal processing schemes for efficient processing of speckles in the reflected probe beam. Recently, an alternative approach to the problem of uneven, rough and poorly reflecting surfaces has been proposed, in which the optical akinetic sensor is positioned in the air near the measuring surface and receives acoustic waves leaking from the sample in the air. Recent advances in the Sagnac architecture have resulted in a flexible system with the improved both sensitivity and stability, and capable of quick optimization of the detection range from a few MHz to GHz. The use of the signal diversity principle through creating multiple independent beams significantly reduced the probability of signal drop-outs in the optical heterodyne detection. These systems have found many practical applications, and most of them have been applied commercially, giving a broad range of options for optical sensing of US vibrations.

One of the most significant problems to be solved in optical detection systems is the long inspection time, especially in the case of multi-dimensional imaging. One possible solution to this problem is to use a parallel detection scheme. Several examples of such systems have been presented, including multi-beam systems [81], [82] or holography [83], [84]. The existing commercial multi-point vibrometers offer the possibility of using up to 48 channels simultaneously [85]. However, this system is limited to a bandwidth of up to 100 kHz. The prototype of a multi-beam laser Doppler vibrometer using six channels in an integrated photonic circuit is a compact device which was reported to be capable of measuring ultrasonic waves in the kHz range [82]. In most US NDT applications, three-dimensional imaging problems are still most often performed using single-beam detectors, which means long scanning time. One of the problems on the way towards the development of multi-channel (or array) optical detectors is the detector’s cost. Each optical detector typically requires expensive components (light source, optical elements, optical fibers and fiber-optic components, detectors, and electronics), making the total cost significant. Additional channels require duplication of components except for a few that can be used for all channels. Thus, the cost of the array will be almost proportional to the number of channels.

A possible solution to the problem of the long inspection time is to use a faster screening method for the initial examination followed by a high-resolution localized imaging using LUT. In the case of thin-walled structures, full-field Lamb wave imaging has been proven to be effective for damage detection using mapping algorithms such as local wavenumber estimation [86], [87], [88], [89]. Although Lamb waves lack the resolution and sensitivity of high frequency LUS, they can be measured using commercial scanning vibrometers with the possibility of using non-contact laser excitation [86], [89]. An example of using full-field local wavenumber imaging in combination with LUT in a multi-resolution approach was presented by [90] to detect and characterize damage in a composite sample of complex shape. At the first stage, the structure was scanned using a commercial vibrometer with signal excitation by a piezoelectric transducer. In the second step, regions of interest determined using local wavenumber maps were scanned using LU excitation with a Sagnac interferometer on receive, obtaining high resolution depth-resolved images of defects. The disadvantage of using different inspection modalities is the cost of individual pieces of equipment.

The need to reduce scanning time in laser ultrasound has led to alternative approaches based on line- of full-field detection. While existing systems using parallel detection are still at an early stage of development and are mainly suited for low-frequency applications with low sensitivity, technological advances in high-speed cameras and electronics might change the situation in future. Therefore, we reference to these methods even though they have not yet demonstrated clear advantages and reliability in NDT field applications. For example, systems based on holography have already been demonstrated for measuring low frequency vibrations from the entire sample surface [83], [84].

An interesting approach was recently proposed in Optical Coherence Tomography (OCT). A conventional approach used for imaging objects with OCT for years was in fast (currently up to a few MHz) scanning of a focused beam over the sample area [91]. Forming a cylindrically focused probe beam as an alternative to the spherically symmetric [92] allows to create a line-scan detection over the sample which dramatically increases the imaging speed. As a tradeoff, the increased imaging rate of the line-scan OCT leads to the reduced quality of OCT images affected, for instance, by the crosstalk between points within the detecting line. Based on the line-scan OCT, detection of slow-propagating shear waves in biological tissues was recently demonstrated with use of a line-field Optical Coherence Elastography (OCE) in the frequency range of a few kHz [93]. Unfortunately, the approach demonstrated in line-field OCE is limited by the rate of line-scan cameras, i.e., by about 200 kHz, which defines the maximum frequency for the detection of US signals. When the rate of line-scan cameras reaches 5–10 MHz, the approach proposed in OCE will most probably be extended to laser ultrasonics. In addition, full-field OCT systems were recently demonstrated [94], [95], which can also attract the attention for their use in the US signal detection.

Non-scanning detection of surface waves in the MHz frequency range was demonstrated for a metal plate in Ref [96] with the full field photorefractive interferometry. Instead of using the conventional focused probe beam scanning over the sample surface, the medium was probed with a wide (16 mm in diameter) beam. The reflected probe light was collected with a system of imaging lenses to obtain an image of the sample surface in the volume of photorefractive crystal (Bi_12_SiO_20_), where it was mixed with the reference beam. Their overlap established a refractive index grating which caused part of each of the beams to diffract towards the other beam. Thus, interference occurred between the weak diffracted beam and the strong transmitted beam, resulting in a heterodyning effect. This effect converted changes in optical phase difference between the overlapping beams induced by the surface motion of the sample into intensity differences of the overlapping beams. The intensity modulations were recorded by a CCD camera. However, unlike the OCE approach which allows propagating waves using a single excitation [93], the full-field photorefractive interferometry in the megahertz range must use a stroboscopic approach. Thus, multiple pump laser firings are required to record the entire wavefield. The physical limitation here is a long response time ($\tau_{R}=6.25\mu s$) of the photorefractive crystal. In addition, CCD cameras do not allow a MHz rate image readout. Another limitation of this method is the sample surface quality, which will affect the projection of its image into the photorefractive crystal.

To address the problem of the sample surface quality in full field imaging, a simple non-scanning speckle-based approach was proposed [96]. Indeed, surface vibration caused by US signals can be computed by calculating the contrast of the reflected speckle pattern [97] generated by a probe laser beam. The speckle pattern can be created by a diffuser positioned in the probe beam. In Ref. [96], the diffused beam was projected to a surface of a glass cuvette with a light-absorbing object inside which was irradiated with nanosecond pump laser pulses (photoacoustic geometry). A temporal resolution of 1 μs was achieved which allowed to obtain an image resolution of about 1 mm. Although the application of this method was not yet in NDE, further progress in high-speed cameras may lead to the development of simple and robust speckle-based optical detectors, at least for recording low-frequency vibrations.

Similar principle is exploited in laser shearography [98], in which a special shearing element is added to the system to produce a coherent superposition of two laterally displaced CCD images (shearogram or interferogram of an object wave with the sheared object wave as a reference wave) of the sample surface in the image plane. Loading induces the deformation of the sample surface. Typical loadings may be mechanical, acoustical, or thermal and may be applied in a static or dynamic way.

Another key point that would radically change the transfer of remote LUS methods to field use would be the ability to inspect parts and objects of complex shape. This problem does not have a universal solution with existing NDT methods. Indeed, though small parts can be imaged in X-ray tomographs [99], the inspection of large-scale objects is hardly possible with this technique. Immersion UT techniques and water jet-coupling UT systems [100] are also limited in their applications. LUT methods do not require coupling and require only a single-sided access to the object under study. However, the LUT faces difficulties in the probe beam alignment, which is the main problem that needs to be solved.

Indeed, the SNR in optical detection is directly proportional to the amount of light collected from the sample surface. As discussed above, industrial grade surfaces are typically rough, which require a high numerical aperture optics to efficiently collect the reflected light. On the other hand, there is a trade-off between the numerical aperture of the imaging lens and the depth of field of detection. This makes confocal optical detectors sensitive to both angular and axial misalignment.

An approach based on the diffuse optical reflection created by surface roughness has been demonstrated by Tecnatom using a confocal Fabry-Perot interferometer [101]. Thus, the detection of LU signals was shown even at large angles to the detection surface normal. Unfortunately, this approach leads to dramatic loss of the detection sensitivity, which makes it effective only when using a pump laser pulse energy in the Joules range and probe optical power level in the range of Watts. This induces multiple problems related to safety, material damage, bulkiness of the LUT system and its extremely high cost.

In contrast to the approach described above, an interesting distance and angle correction system (DACS) was proposed by Canfield et al. [102]. It uses two additional light sources, which, when reflected from the target, enabled high-speed mechanisms for the independent compensation of distance and angle misalignments of the Sagnac detection head relative to the surface. DACS has been shown to perform consistently across a variety of composite surfaces, while providing ± 2° auto angular and ± 2 mm axial autocorrection with a maximum realignment time of 100 ms. Although DACS has been shown to work with the Sagnac interferometer, it can easily be adapted to other interferometers. The range of angular and distance corrections provided by DACS does not allow LUT scanning of samples of arbitrary shapes and completely solving the detection beam alignment problem in LUT systems, but this approach can be used in combination with fast robotic scanning over the inspection surface with feedback for an approximate alignment. The tremendous recent progress in robotics, electronics and automatic vision systems give reason to hope that the problem of alignment in LUT will be accomplished soon.

## Declaration of Competing Interest

The authors declare the following financial interests/personal relationships which may be considered as potential competing interests: Ivan Pelivanov is a co-founder and shareholder of LuxSonics Inc. Other authors declare no conflict of interests.

## Data Availability

No data was used for the research described in the article.
